# Xylosyltransferase-II deficiency rewires innate immune signaling and destabilizes polarization in human macrophages

**DOI:** 10.3389/fimmu.2026.1850248

**Published:** 2026-06-26

**Authors:** Alina Witt, Thanh-Diep Ly, Matthias Kühle, Cornelius Knabbe, Isabel Faust-Hinse

**Affiliations:** 1 Herz- und Diabeteszentrum Nordrhein-Westfalen, Institut für Laboratoriums- und Transfusionsmedizin, Universitätsklinik der Ruhr-Universität Bochum, Bad Oeynhausen, Germany; 2 Herz- und Diabeteszentrum Nordrhein-Westfalen, Medizinische Fakultät OWL (Universität Bielefeld), Bad Oeynhausen, Germany

**Keywords:** inflammation, macrophages, NF-κB, osteoclast, polarization, spondylo-ocular syndrome, xylosyltransferase-II

## Abstract

Xylosyltransferase-II (XT-II), encoded by *XYLT2*, catalyzes the rate-limiting initial step of proteoglycan biosynthesis. Pathogenic mutations in *XYLT2* cause spondylo-ocular syndrome (SOS), a rare disorder characterized by severe primary osteoporosis, skeletal dysplasia, and additional systemic manifestations. Although dysregulated macrophage polarization has been linked to bone remodeling disorders such as osteoporosis, which are associated with an imbalance toward pro-inflammatory and osteoclastogenic signaling, the role of XT-II in macrophage biology remains unexplored. In the present study, siRNA-mediated knockdown of *XYLT2* was performed in human primary macrophages across three polarization states (M0, M1, M2) to investigate the impact of XT-II deficiency on macrophage polarization and inflammatory signaling. At the phenotypic level, *XYLT2* deficiency destabilized M2 macrophage identity, as shown by significant reductions in M2-associated marker expression, including interleukin 1β, interleukin 6, CD163, and CD206, and impaired phagocytic capacity. A paradoxical reduction of pro-inflammatory markers alongside the induction of M2-associated features was observed in M1 macrophages, indicating a broader disruption of polarization boundary maintenance. The *XYLT2* deficiency also promoted pro-inflammatory activation in unpolarized M0 macrophages. Cytokine profiling revealed a predominantly pro-inflammatory secretory shift, with exceptional induction of CXCL10 across all polarization states. At the signaling level, *XYLT2* knockdown resulted in a reciprocal shift, characterized by suppression of NF-κB pathway components and nuclear p65 translocation alongside the constitutive activation of STAT1 and STAT3. Transcriptome-wide profiling by bulk mRNA sequencing confirmed a conserved interferon-associated gene expression program across all polarization states, with significant enrichment of innate immune sensing, JAK-STAT, and cytokine signaling pathways among upregulated genes. In summary, these findings demonstrate that XT-II fulfills an important role in maintaining macrophage polarization and that its deficiency induces a reprogramming of pro-inflammatory signaling, extracellular matrix remodeling, and cellular interactions in bone-relevant cell types. This is associated with transcriptional changes linked to osteoclast differentiation and altered bone-associated immune signaling, suggesting that *XYLT2* deficiency may contribute to impaired bone homeostasis and providing new insights into the pathomechanisms of SOS.

## Introduction

Macrophages are highly plastic, innate immune cells with the capacity to dynamically adapt their functional programs in response to environmental stimuli. They originate from the lineage of hematopoietic stem cells located in the bone marrow, which give rise to circulating monocytes (1). As immediate precursors of macrophages, monocytes circulate in the blood and, upon inflammatory or homeostatic signals, migrate into peripheral tissues, where they differentiate into macrophages (2). Macrophages can adopt different polarization states in response to microenvironmental cues due to their phenotypic plasticity. The subtypes are basically divided into: unpolarized and inactive (M0), classical-activated and pro-inflammatory (M1), and alternatively activated and anti-inflammatory (M2) (3, 4). M1 macrophages are typically induced *in vitro* by interferon-γ (IFNγ) and lipopolysaccharide (LPS). They are characterized by a high expression of interleukin-1β (IL1β), interleukin-6 (IL6), and tumor necrosis factor-α (TNFα), and functionally promote antimicrobial responses, production of reactive oxygen and nitrogen species, and sustained pro-inflammatory signaling (5–7). The signaling via the transcription factor nuclear factor ‘kappa-light-chain-enhancer’ of activated B-cells (NF-κB) is one of the major pathways governing pro-inflammatory M1 macrophage activation (8). The NF-κB/Rel family comprises five members: p50 (NF-κB1), p52 (NF-κB2), p65 (RelA), c-Rel, and RelB. Upon lipopolysaccharide stimulation via Toll-like receptor 4 (TLR4), inhibitory IκB proteins are degraded, enabling nuclear translocation of the canonical NF-κB p65/p50 heterodimer and induction of a broad pro-inflammatory program (8–12). Interferon-γ contributes an additional, complementary layer of activation through the Janus kinase/signal transducers and activators of transcription 1 (JAK-STAT1) axis, inducing the expression of IFN-stimulated genes (ISGs) that amplify and sustain the inflammatory state (13, 14). Notably, STAT1 and NF-κB pathways are functionally interconnected: activated STAT1 can attenuate NF-κB-driven transcription, and the transcriptional balance between these programs defines the stability of macrophage polarization states (15–23). By contrast, M2 macrophages arise *in vitro* in response to IL4 and are associated with tissue repair, extracellular matrix (ECM) remodeling, and resolution of inflammation, characterized by expression of mannose receptor I (CD206) and hemoglobin scavenger receptor (CD163), and secrete anti-inflammatory mediators, such as IL10 (19–21). M2 polarization is driven by IL4/STAT6 signaling, whereas IL-10 activates STAT3, promoting anti-inflammatory programs and attenuating NF-κB activity (17, 22). The functional outcome of STAT3 activation is stimulus-dependent, however, as pro-inflammatory cytokines such as IL-6 can engage the same pathway to drive opposing gene programs (23), illustrating that macrophage polarization represents a dynamic continuum rather than a binary state based on their environment (24–26).

In addition to their role as inflammatory effectors, macrophages maintain tissue homeostasis through the phagocytic clearance of pathogens, apoptotic cells, and extracellular debris, as well as through the active support of ECM remodeling following tissue damage (27). The ECM itself, however, is not merely a passive scaffold, but actively shapes macrophage signaling by modulating cytokine availability, receptor clustering, and growth factor gradients, with proteoglycans (PGs) playing a particularly important role as regulators of cell-matrix communication (28). Through their glycosaminoglycan (GAG) side chains, PGs regulate the sequestration, presentation, and diffusion of cytokines and growth factors within the tissue microenvironment and thereby critically influence inflammatory signaling pathways (29). Consequently, impaired PG biosynthesis may alter macrophage activation states and destabilize polarization programs by disrupting ECM-dependent immune regulatory mechanisms. In tissues with a high dependence on continuous ECM remodeling, such as bone, this reciprocal interaction between macrophages and the ECM positions macrophages as important regulators of bone homeostasis. Both polarization states actively contribute to this process: M2 macrophages promote osteogenic differentiation and inhibit osteoclast differentiation through secretion of IL10 and transforming growth factor β, while M1 macrophages, when transiently activated, support early bone repair through angiogenesis and mesenchymal stem cell recruitment (30). However, persistent M1-dominated inflammation shifts this balance. Pro-inflammatory cytokines, such as TNFα, IL6, and IL1β, promote RANK-L expression, leading to osteoclast hyperactivation and increased bone resorption, while simultaneously impairing osteogenesis (31, 32). Diseases characterized by this chronic imbalance, such as osteoporosis and rheumatoid arthritis, are, thus, also closely linked to dysregulated macrophage polarization (33, 34).

The Spondylo-ocular syndrome (SOS; OMIM #605822), a rare autosomal recessive disorder, is characterized by severe osteoporosis and skeletal dysplasia, alongside ocular, cardiac and hearing impairment involvement (35, 36). It is caused by pathogen mutations in *XYLT2*, which encodes xylosyltransferase II (XT-II) (37, 38). XT-II catalyzes the initial and rate-limiting step of PG biosynthesis by transferring xylose from UDP-xylose onto specific serine residues of core proteins, thereby initiating the assembly of GAG chains and enabling the formation of structurally and functionally intact PGs required for ECM organization and cell-matrix signaling (38). In addition to XT-II, in humans a second isoform was describes, the XT-I, which is encoded by *XYLT1*. Both isoforms perform the same catalytic reaction, yet they differ in their expression profiles, functional contexts and associated disease manifestations. *XYLT2* is ubiquitously expressed and constitutes the dominant isoform in the majority of human cell types, while *XYLT1* shows a more restricted, tissue-specific expression pattern (39). Furthermore, pathogen *XYLT1* mutations cause Desbuquois dysplasia type 2 (DBQD2; MIM #608124), a distinct skeletal dysplasia, characterized by facial deformities, growth retardation, and short long bones (40). Both diseases, SOS and DBQD2, are classified as GAG linkeropathies, underscoring the non-redundant role of each isoform despite their shared catalytic function (41). Given the involvement of PGs in ECM organization and cytokine signaling, impaired *XYLT2*-dependent PG biosynthesis may not only compromise structural tissue integrity but could also alter immune-regulatory microenvironments that govern macrophage activation and bone homeostasis (42). This is particularly relevant in SOS, where severe osteoporosis occurs in the context of a systemic ECM biosynthesis defect. In line with a potential role of XT in macrophage biology, previous work from our group demonstrated that *XYLT1* is upregulated in M2-polarized macrophages, identifying *XYLT1* as a polarization-responsive gene. However, siRNA-mediated knockdown of *XYLT1* had no detectable effect on macrophage polarization, indicating that XT-I is dispensable for maintaining macrophage function (43).

However, it remains unclear which role XT-II fulfills in macrophage biology and contributes to the regulation of macrophage polarization and inflammatory signaling, and whether its deficiency may thereby influence disease-relevant processes such as bone homeostasis and osteoclast differentiation and activity in the context of SOS. Therefore, the present study investigates the impact of *XYLT2* deficiency on macrophage polarization and inflammatory signaling in human primary macrophages.

## Materials and methods

### Materials

All reagents, consumables, and buffers required for the negative selection of monocytes were purchased from Miltenyi Biotec (Bergisch Gladbach, Germany), including the PAN monocyte isolation kit, MACS BSA stock solution, autoMACS rinsing solution, as well as the MACS separator and LS columns. Recombinant cytokines and growth factors used for macrophage differentiation and polarization [macrophage colony-stimulating factor (M-CSF), IL4, and IFNγ] were obtained from PeproTech (Hamburg, Germany), while LPS, derived from *Escherichia coli* serotype O111:B4, was supplied by Sigma-Aldrich (St. Louis, MO, USA). Lipofectamine 2000, 1× Opti-MEM I reduced-serum medium, *XYLT2*-specific siRNA (ID 112166), and a non-targeting control siRNA (Silencer Negative Control #1) were sourced from Thermo Fisher Scientific (Waltham, MA, USA).

### Human samples

Buffy coats (approximately 50 mL) from healthy, anonymous blood donors were obtained from the Institute for Laboratory and Transfusion Medicine, Heart and Diabetes Center NRW, University Hospital of the Ruhr University Bochum (Bad Oeynhausen, Germany). The study protocol was reviewed and approved by the local Ethics Committee of the Heart and Diabetes Center NRW, Department of Medicine, Ruhr University Bochum (approval number 2022_916_2; November 12, 2025).

### Isolation of peripheral blood mononuclear cells from buffy coats

The isolation of PBMCs from buffy coats was performed as described previously (43). The PBMCs were isolated from buffy coats by density gradient centrifugation using Ficoll-Paque PLUS (density 1.078 g/mL; Thermo Fisher Scientific, Waltham, MA, USA). Buffy coats were diluted 1:1 with pre-warmed Dulbecco’s phosphate-buffered saline (1x PBS) prior to separation. Aliquots of the diluted samples were carefully layered onto the density gradient medium and centrifuged (760 x g, 20 min, brake off). Following centrifugation, the mononuclear cell layer was collected from the interphase and transferred into fresh tubes. Harvested cells were washed repeatedly with 1x PBS by centrifugation (350 x g, 8 min, brake on). An additional low-speed centrifugation step was performed (200 x g, 10 min, brake on) to further reduce platelet contamination. Residual erythrocytes were removed by incubating the cells in Red Cell Lysis Buffer (PromoCell, Heidelberg, Germany) for 15 min at room temperature. The lysis reaction was terminated by adding 1x PBS to a final volume of 50 mL, followed by centrifugation (200 x g, 10 min, brake on). The PBMCs were pooled and resuspended in wash buffer for differential blood cell analysis using a Sysmex XN-1000 hematology analyzer (Sysmex Corporation, Kobe, Japan). After an additional centrifugation step, cells were resuspended in complete Roswell Park Memorial Institute (RPMI)-1640 medium supplemented with 10% (v/v) fetal bovine serum (Biowest, Nuaillé, France), 2 mM L-glutamine (PAN Biotech, Aidenbach, Germany) and 1% (v/v) penicillin–streptomycin–amphotericin mix (100×; PAN Biotech). The PBMCs were adjusted to the required concentration and either used immediately for downstream applications or cryopreserved. Regarding the latter, cell suspensions were mixed 1:1 (v/v) with freezing medium containing 20% (v/v) dimethyl sulfoxide (Carl Roth, Karlsruhe, Germany) in fetal bovine serum, transferred to cryovials, gradually cooled to -80 °C within 24 h, and subsequently stored in liquid nitrogen.

### Isolation of monocytes from PBMCs

Monocytes were isolated from PBMCs using the PAN Monocyte Isolation, according to the manufacturer’s protocol. The PBMCs were incubated with FcR Blocking Reagent and a biotin-conjugated antibody cocktail (10 µL per 1 × 10^7^ total cells) for 5 min at 4 °C. Subsequently, anti-biotin magnetic microbeads (20 µL per 1 × 10^7^ total cells) were added, followed by incubation for 10 min at 4 °C. Monocytes were enriched by negative magnetic separation using LS columns with a total binding capacity of 1.5 x 10^8^ cells per column. After isolation, the monocyte purity was assessed using an automated hematology analyzer (Sysmex XN-1000, Sysmex Corporation, Kobe, Japan). Only cell preparations with a monocyte content exceeding 85% were used for subsequent experiments.

### Cultivation and polarization of human primary macrophages

Negatively selected monocytes were cultured under standardized aseptic conditions (37 °C, 5% CO_2_) in complete RPMI-1640 medium supplemented with 10% (v/v) fetal bovine serum (Biowest, Nuaillé, France), 2 mM L-glutamine (PAN Biotech, Aidenbach, Germany), 1% (v/v) penicillin–streptomycin–amphotericin mix (100×; PAN Biotech) and 50 ng/mL M-CSF for macrophage differentiation. Cells were seeded with a density of 1 × 10^5^ per cm^2^ and cultured for eight days, with the day of seeding defined as day 0. On day 2, half of the culture medium was replaced with fresh complete medium containing M-CSF. Macrophage polarization was performed on day 6 by replacing the culture medium with fresh RPMI-1640 medium supplemented with IFNγ (50 ng/mL) and LPS (10 ng/mL) for M1-like macrophages, or IL4 (20 ng/mL) for M2-like macrophages. Cells were maintained in medium containing M-CSF without additional stimuli for the generation of non-polarized (M0) macrophages. Cells were polarized for 48 h before macrophages and culture supernatants were collected for downstream analyses on day 8.

### Transfection of human primary macrophages

The siRNA-mediated gene silencing was performed in adherent primary human macrophages on day 5 of differentiation. Prior to transfection, cells were gently washed with pre-warmed 1x PBS to remove any nonadherent cells and residual antibiotics. Macrophages were then supplied with fresh antibiotic-free complete RPMI-1640 medium containing 75 ng/mL M-CSF. Depending on the culture format, 1 mL of medium was added per well of a six-well plate, 500 µL per well of a 12-well plate, or 100 µL per well of a removable 8-well chamber glass slides (Ibidi, Gräfelfing, Germany). siRNA-lipid complexes were prepared for each transfection condition using Lipofectamine 2000 and Opti-MEM, according to standardized conditions. A 5 µM siRNA stock (*XYLT2*-specific siRNA (ID 112166) or a non-targeting control siRNA (Silencer Negative Control #1)) solution prepared in RNase-free water was diluted in Opti-MEM to obtain a final siRNA concentration of 50 nM per well. To address knockdown specificity was additionally assessed using an independent XYLT2-targeting siRNA (ID 112168, Thermo Fisher Scientific, Waltham, MA, USA; data not shown). Furthermore, the employed Silencer Pre-designed siRNAs incorporate manufacturer-validated sequence optimization and off-target filtering strategies to improve silencing specificity. In parallel, Lipofectamine 2000 was diluted 1:50 (v/v) in pre-warmed Opti-MEM and incubated for 5 min at room temperature. The diluted siRNA and Lipofectamine 2000 solutions were subsequently combined at a 1:1 (v/v) and allowed to complex for 15 min at room temperature. The resulting solutions, corresponding to one-third of the final culture volume, were added dropwise to the macrophage cultures. Cells were incubated under standard culture conditions (37 °C, 5% CO_2_) for 6 h. The cells were washed with 1x PBS and supplied with complete RPMI-1640 medium for overnight recovery. The polarization of transfected macrophages was performed on day 6, as described above.

### Gene expression analysis

Total RNA was isolated from human primary macrophage lysates, following the manufacturer’s protocol from the NucleoSpin RNA Kit (Macherey-Nagel, Düren, Germany). The RNA concentration and purity were determined using a NanoDrop 2000 spectrophotometer (Peqlab, Erlangen, Germany). Only RNA samples with A260/A280 and A260/A230 ratios between 1.9 and 2.2 were used for downstream applications. An amount of 1 µg of total RNA was reverse-transcribed using GoScript Reverse Transcription Mix with Oligo(dT) (Promega, Madison, USA) for cDNA synthesis. The resulting cDNA was diluted 1:19 with nuclease-free water prior to quantitative real-time PCR (qRT-PCR). Each qRT-PCR reaction contained 2.5 µL of diluted cDNA, 0.25 µL of forward and reverse primers (25 µM; Biomers, Ulm, Germany), 5 µL SYBR Green I Master Mix (Roche, Penzberg, Germany), and nuclease-free water to a final volume of 10 µL. The qRT-PCR was performed using standard cycling conditions, followed by a melting curve analysis to verify the amplification specificity. All reactions were run in technical triplicate. Relative mRNA expression levels were calculated according to the efficiency-corrected model described by Pfaffl et al. (44). Data normalization was performed using a reference gene index expression comprising succinate dehydrogenase complex flavoprotein subunit A (*SDHA*), ribosomal protein L13a (*RPL13A)* and ß2-microglobulin (*B2M*). Primer sequences and the protocol for qRT-PCR used for gene expression analysis are listed in **Supplementary Tables 1**, **S2**.

### mRNA-sequencing

Total RNA from human primary macrophage lysates was isolated, as described above. The RNA concentration was determined using an AccuBlue^®^ Broad Range RNA Quantification Kit (Biotium, Fremont, CA, USA). The mRNA sequencing, including quality control, RNA library preparation, and sequencing itself was performed by Alithea Genomics SA (Épalinges, Switzerland), using the MERCURIUS™ BRB-seq platform on an Illumina sequencer (San Diego, CA, USA). The RNA from four independent donors per polarization state (M0, M1, M2) and condition (siNC, si*XYLT2*) was analyzed. All bioinformatic analyses were performed in R (version 4.3.3) using the DESeq2 package (45). A separate DESeq2 object was constructed from the raw UMI count matrix for each macrophage polarization state, and a multifactor design was used to test for differences in the gene expression between si*XYLT2* and siNC (condition), while accounting for sample pairing by cell donors (donors) using a general linear model (~ donor + condition). Low-expression genes were excluded by retaining only those with a minimum count of 4 in at least three samples. Differentially expressed genes (DEGs) were identified using the Wald test, and log2 fold change (LFC) estimates were regularized using the apeglm shrinkage method (46). Genes with an adjusted p-value < 0.05 and an absolute LFC ≥ 0.5 were considered differentially expressed. Gene symbols were annotated using the org.Hs.eg.db package (47). Pathway enrichment analysis was performed using ShinyGO (version 0.85.1) (48) by uploading the list of significant DEGs and mapping them to human KEGG signaling pathways at a false discovery rate (FDR) threshold of 0.05.

### Cell characterization with flow cytometry

Phenotypic characterization of human primary macrophages was performed by flow cytometry using a BD FACSCanto II flow cytometer (BD Biosciences, Franklin Lakes, NJ, USA). Expression of TNFα-PerCP-Cy5.5 and CD206-APC-H7 (BD Bioscience, Franklin Lakes, NJ, USA) was assessed using fluorochrome-conjugated monoclonal antibodies. Regarding cell collection, macrophages were detached by incubation with 1 mL accutase for 5–8 min at 37 °C. Cell suspensions were centrifuged at 350 x g for 8 min and washed with 1x PBS. Cells were fixed in fixation buffer (R&D Systems, Minnesota, USA) for 10 min at room temperature, with intermittent mixing, followed by washing with 1x PBS. Fc receptor blocking was performed by incubating cells with Fc block (100 µg/mL; BD Biosciences) for 25 min at room temperature. An amount of 0.25 × 10^6^ cells were incubated for 45 min at 4 °C in the dark with fluorophore-conjugated antibodies using manufacturer-recommended dilutions in permeabilization buffer (R&D Systems, Minnesota, USA) for antibody staining. Following incubation, cells were washed with 1x PBS and resuspended in FACS buffer (1:20, autoMACS Rinsing Solution: MACS BSA Stock Solution, Miltenyi Biotec, Bergisch Gladbach, Germany) for acquisition and 10,000 events were analyzed per condition. Flow cytometry data were acquired using BD FACSCanto software and analyzed with Kaluza Analysis Software (Version 2.3, Beckman Coulter, Brea, CA, USA). Median fluorescence intensity (MFI) values were calculated for each marker.

### Cytokine quantification via immunoassay

Interleukin-6 concentrations in cell culture supernatants of polarized macrophages were quantified using the automated immunoanalyzer Cobas e411 (Roche, Basel, Switzerland). They were normalized to the corresponding DNA content of each sample. Genomic DNA was isolated using the NucleoSpin Blood Extraction Kit (Macherey-Nagel, Düren, Germany), following the manufacturer’s protocol. In addition, cytokine profiles in cell culture supernatants were analyzed using a Human Cytokine Array Kit (R&D Systems, Minneapolis, MN, USA), according to the manufacturer’s instructions. Chemiluminescent signals were detected using a Fusion SL imaging system (Vilber Lourmat, France) and quantified utilizing ImageJ software (version 1.54p) (49). Signal intensities were normalized to the reference spots provided on the membrane, and results for si*XYLT2*-treated samples were expressed relative to the siNC control, yielding a relative change in cytokine abundance.

### Phagocytosis assay

Phagocytic activity of human primary macrophages was assessed using pHrodo BioParticles Conjugates for Phagocytosis (Thermo Fisher Scientific, Waltham, MA, USA). Macrophages were cultured in 12-well plates and differentiated as described above. The culture medium was replaced with fresh complete RPMI-1640 medium supplemented with phagocytosis particles at a dilution of 1:10 on day 8 of cultivation. Cells were incubated for 3 h at 37 °C under atmospheric conditions without CO_2_. Following incubation, the medium was removed and replaced with complete RPMI-1640 medium containing Calcein AM (1:1000, Sigma-Aldrich, St. Louis, MO, USA)) and 4′,6-diamidino-2-phenylindole (DAPI) (1:1000, Thermo Fisher Scientific, Waltham, MA, USA) to stain viable cells and nuclei, respectively. Cells were incubated for an additional 30 min at 37 °C without CO_2_. After staining, macrophages were washed with 1x PBS and overlaid with complete RPMI-1640 medium for imaging. Images were acquired using a Keyence BZ-X810 fluorescence microscope (Keyence, Osaka, Japan). Five representative fields were analyzed for each donor and experimental condition. Quantitative image analysis was performed using ImageJ software (version 1.54p) (49). Nuclei were identified for each image based on DAPI staining and used to determine cell numbers. Phagocytosed particles were quantified by thresholding the fluorescence signal of the particles and measuring the particle-positive area per image. Phagocytic activity was expressed as a particle-positive area normalized to the number of nuclei, yielding a relative phagocytic activity.

### Immunofluorescence microscopy

Polarized macrophages were fixed with 4% (w/v) paraformaldehyde (Roti Histofix, Carl Roth, Karlsruhe, Germany) for 20 min at room temperature. Where indicated, macrophages were additionally stimulated with TNFα (20 ng/mL), IFNγ (50 ng/mL), or IL6 (50 ng/mL) for 30 min prior to fixation to assess stimulus-induced signaling responses. Following the fixation, cell monolayers were washed with 1x PBS and stored in PBS at 4 °C until further processing. All subsequent staining steps were carried out at room temperature and cells were washed three times with 1x PBS after each incubation step. Negative control samples incubated only with secondary antibody were included for each macrophage polarization state. Cells were permeabilized with ice-cold 100% (v/v) methanol for 10 min at -20 °C where required by the antibody manufacturer. Nonspecific binding was blocked by incubation with blocking buffer containing 5% (v/v) normal goat serum (Sigma-Aldrich, St. Louis, MO, USA) and 0.3% (*v*/*v*) Triton X-100 (Sigma-Aldrich, St. Louis, MO, USA) in 1x PBS for 1 h. Primary antibody incubation was performed overnight at 4 °C using monoclonal rabbit anti-human antibodies against NF-κB p65 #8242, pSTAT1 #9167, or pSTAT3 #9145 (each diluted 1:200; Cell Signaling Technology, MA, USA) prepared in antibody dilution buffer (1% (w/v) bovine serum albumin and 0.3% (v/v) Triton X-100 in 1x PBS). Cells were incubated for 2 h in the dark with a fluorophore-conjugated secondary antibody (goat anti-rabbit IgG, Alexa Fluor 555; 1:800; Abcam, Cambridge, UK) diluted in antibody dilution buffer. The nuclei were counterstained with DAPI (1:1000 in 1x PBS; Thermo Fisher Scientific, Waltham, MA, USA). Coverslips were mounted using Roti-Mount Aqua mounting medium (Carl Roth, Karlsruhe, Germany). Immunofluorescence images were acquired using a Keyence BZ-X810 fluorescence microscope (Keyence, Osaka, Japan). To analyze the nuclear translocation, five representative fields were analyzed for each donor and experimental condition. Quantitative image analysis was performed using ImageJ software (version 1.54p) (49). Nuclear regions were manually delineated, and fluorescence intensities were measured and normalized to the respective nuclear area.

### Mass spectrometric XT activity assay

The relative quantification of the intracellular XT-I/-II activity from cell lysates was performed using a selective mass spectrometric enzyme activity assay, following a procedure published previously (50). This relative XT-I/-II activity measurement relies on XT-I/-II-catalyzed xylose incorporation into an XT-I/-II-selective acceptor peptide after a defined period. Cell lysates for intracellular XT quantification were obtained by incubating the macrophage monolayer with Nonidet P-40 Substitute (Roche, Basel, Switzerland) containing lysis buffer (500 μL); the samples were centrifuged (11,000 x *g*, 10 min, room temperature) after harvesting. The supernatant could be stored at -80 °C. The samples were concentrated prior to further analysis using Amicon Ultra centrifugal filter units with a molecular weight cutoff of 10 kDa (Sigma-Aldrich, St. Louis, MO, USA). The reaction mixture consisted of an XT-I or XT-II-specific acceptor peptide (AP1 or AP2, 10 µL, 60 µg/mL), UDP-xylose (10 µL, 80 µg/mL), Mg/Mn solution (2.5 µL, 5 mmol/L), MES buffer (2.5 µL, 25 mmol/L, pH 6.5), and 25 µL of the respective sample. Reactions were incubated at 37 °C for 24 h. All assays were performed in parallel for the acceptor peptides AP1 and AP2. Enzymatic reactions were terminated by heat denaturation at 99 °C for 10 min, followed by centrifugation at 10,000 x g for 10 min at 4 °C. Supernatants (20 µL each) from the AP1 and AP2 reactions were combined and diluted with 60 µL of water prior to analysis. Quantification of xylosylated peptides (AP1-Xyl and AP2-Xyl) was carried out using in-line solid-phase extraction into ultra-performance liquid chromatography tandem mass spectrometry in multi reaction monitoring mode (SPE-UPLC-MS/MS). Chromatographic separation and mass spectrometric detection were performed as described previously. Electrospray ionization was conducted in positive ion mode using a capillary voltage of 2.0 kV, a desolvation temperature of 600 °C, a desolvation gas flow of 900 L/h, and a collision gas flow of 0.15 mL/min. Enzymatic activity was quantified by integration of multiple-reaction monitoring peak areas. Data acquisition and automated analysis were performed using TargetLynx software within the MassLynx platform (Waters, Massachusetts, USA). Results were normalized to protein levels of each sample, determined by BCA assay according to Smith et al. (51).

### Statistical analysis

Unless otherwise stated, all statistical analyses of experimental data were performed using GraphPad Prism (version 10.0; GraphPad Software, San Diego, CA, USA). Statistical comparisons were performed at the level of independent samples, with each donor contributing a single mean value per condition, calculated by averaging across biological and technical replicates prior to analysis. A two-way ANOVA was applied for group comparisons. Data are presented as mean ± standard error of the mean (SEM). Differences with a p-value of 0.05 or less were considered statistically significant. Statistical analyses of mRNA sequencing data are described in the mRNA sequencing section.

## Results

### The siRNA-mediated knockdown of *XYLT2* reduces XT-I and XT-II activity in human primary macrophages

**Figure 1:**
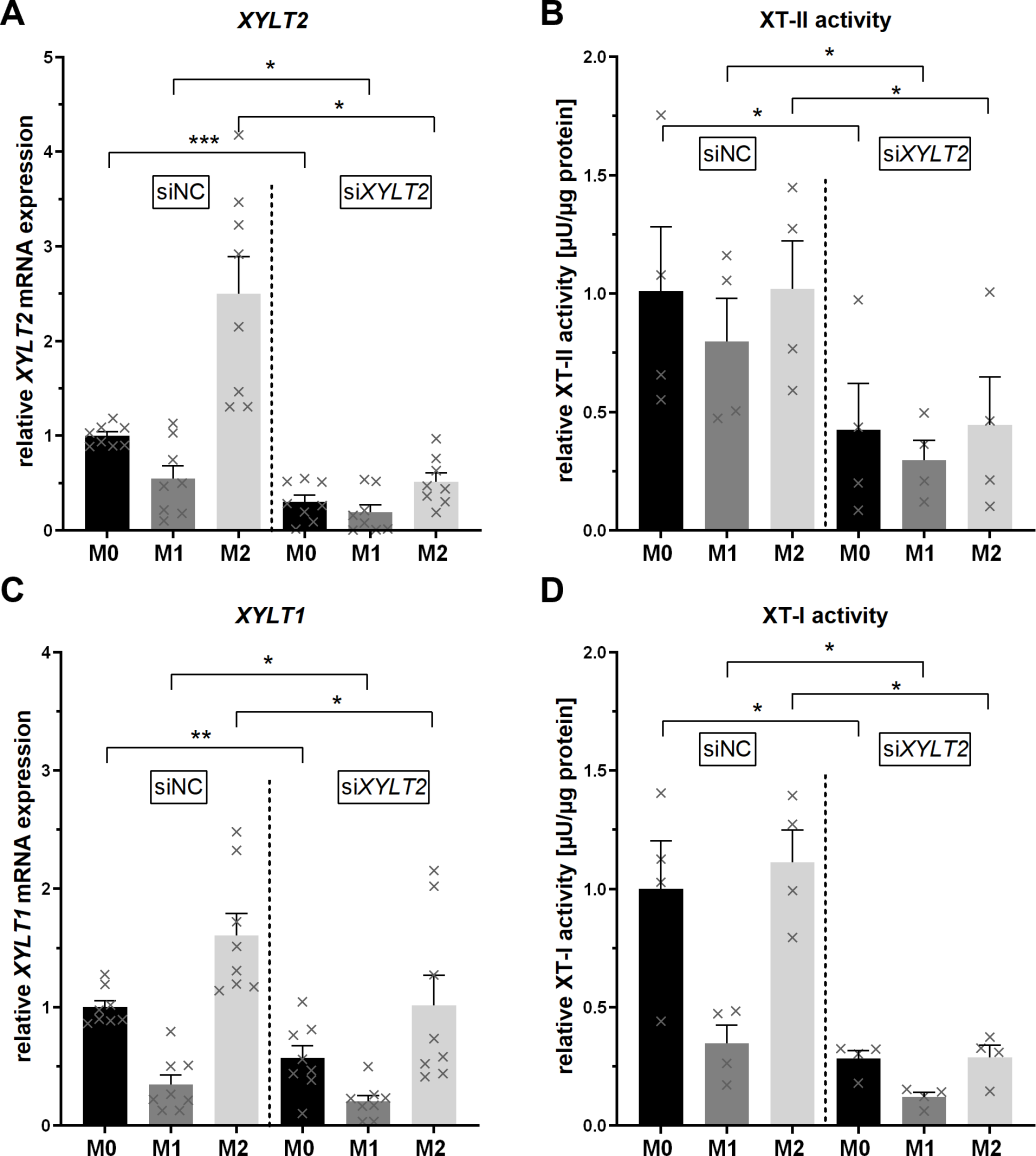
Gene expression analysis of XYLT2 and XYLT1 as well as XT-II and XT-I activity of polarized macrophages after siRNA-mediated XYLT2 knockdown. Negatively selected monocytes were differentiated into macrophages using M-CSF. Macrophages were treated with a non-targeting negative control siRNA (siNC) or a siRNA targeting XYLT2 (siXYLT2) on day 5. On day 6, macrophages were stimulated with IFNγ/LPS (M1), IL4 (M2), or with no additive (M0). Cells were harvested for qRT-PCR analysis (n = 8; 2 biological and 3 technical replicates per condition) or XT-activity assay via mass spectrometry (n = 4; 2 biological and 2 technical replicates per condition) after a polarization time of 48 h. The relative gene expression levels of XYLT2 **(A)** and XYLT1 **(C)** as well as XT-II **(B)** and XT-I **(D)** activity were analyzed. Data are shown as mean ± SEM. Statistical significance was determined using Two-Way-ANOVA: p ≤ 0.05 (*), p ≤ 0.01 (**), p ≤ 0.001 (***).

The siRNA-mediated knockdown of *XYLT2* was performed in primary human macrophages across all three phenotypes to investigate the functional role of XT-II in macrophage biology. Knockdown efficiency was confirmed at both the transcript and enzymatic activity level. The *XYLT2* mRNA expression was significantly reduced in si*XYLT2*-treated macrophages (M0: 70%; M1: 55%; M2: 78%) compared to siNC controls (**Figure 1A**). Notably, *XYLT2* expression under siNC conditions was elevated in M2 macrophages compared to M0 and M1, showing a polarization-dependent regulation of this isoform. In line with the mRNA data, XT-II enzymatic activity was significantly reduced in si*XYLT2*-macrophages (M0: 53%; M1: 58%; M2: 50%) compared to their respective siNC controls (**Figure 1B**). Strikingly, *XYLT1* mRNA expression was also significantly downregulated following *XYLT2* knockdown in all polarization states (**Figure 1C**), mirroring the expression pattern observed for *XYLT2*. The *XYLT1* mRNA expression in si*XYLT2*-M0 macrophages was decreased by 46%, downregulated by 44% in si*XYLT2*-M1 macrophages, and by 41% in si*XYLT2*-M2 macrophages. The *XYLT1* was markedly upregulated in M2 macrophages under siNC conditions, identifying it as a polarization-responsive gene, as previously reported by our group (43). The coordinated downregulation of *XYLT1* upon *XYLT2* knockdown was further reflected at the enzymatic level: XT-I activity was significantly reduced in si*XYLT2*-treated macrophages across all phenotypes (M0: 72%; M1: 65%; M2: 74%) compared to siNC controls (**Figure 1D**). These findings indicate that *XYLT2* knockdown leads to a coordinated reduction of both XT-II and XT-I activity across all polarization states, demonstrating a functional coupling of the two isoforms and establishing XT-II as a key determinant of overall XT capacity in macrophages.

### *XYLT2* knockdown in human macrophages alters macrophage polarization and phagocytic activity

**Figure 2:**
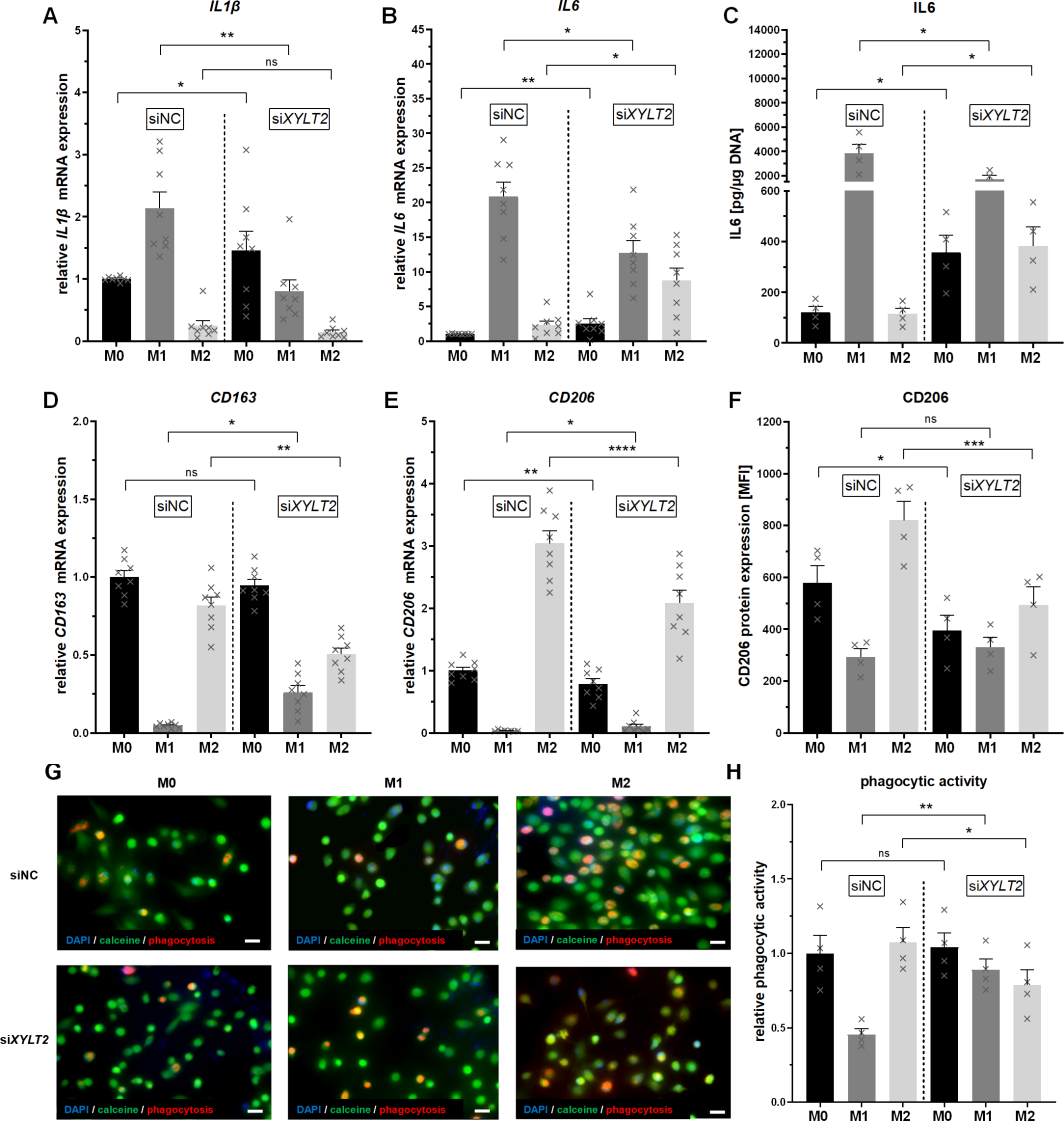
Expression analysis of polarization marker and phagocytic activity of polarized macrophages after siRNA-mediated XYLT2 knockdown. Negatively selected monocytes were differentiated into macrophages using M-CSF. Macrophages were treated with a non-targeting negative control siRNA (siNC) or a siRNA targeting XYLT2 (siXYLT2) on day 5. On day 6, macrophages were stimulated with IFNγ/LPS (M1), IL4 (M2), or with no additive (M0). Cells were harvested for qRT-PCR analysis (n = 8; 2 biological and 3 technical replicates per condition) or protein expression or phagocytic activity (n = 4; 2 biological and 1 technical replicates per condition) after a polarization time of 48 h. The relative gene or protein expression levels of IL1β **(A)**, IL6 **(B)**, IL6 **(C)**, CD163 **(D)**, CD206 **(E)**, and CD206 **(F)** were analyzed. The phagocytic activity was determined using pHrodo™ Red **(E)** coli and analyzed via fluorescence microscopy, exemplary images shown **(G)**, and **(H)** quantification by ImageJ, cells were counterstained using calceine (Live/Dead) and DAPI (nuclei). Scale bar = 10 µm. Data are shown as mean ± SEM. Statistical significance was determined using a Two-Way-ANOVA: ns, not significant; p ≤ 0.05 (*), p ≤ 0.01 (**), p ≤ 0.001 (***), p ≤ 0.0001 (****).

The expression of established M1- and M2-associated marker genes was analyzed across all three polarization states to analyze the impact of *XYLT2* knockdown on macrophage polarization. Light microscopy revealed morphological alterations in si*XYLT2*-treated macrophages relative to siNC controls prior to the molecular analysis: While si*XYLT2-*M1 macrophages appeared morphologically unchanged, si*XYLT2*-M0 and si*XYLT2-*M2 macrophages displayed a more rounded cell morphology compared to their respective siNC controls, reminiscent of the M1 polarization state (**Supplementary Figure 1**). Firstly, the M1-marker was analyzed: Pro-inflammatory cytokine *IL1β* was significantly upregulated 1.5-fold in si*XYLT2*-M0 macrophages compared to siNC controls, while a significant reduction was observed in si*XYLT2*-M1 macrophages of about 61% and a trend toward reduction of about 40% in was detected in *siXYLT2-*M2 macrophages (**Figure 2A**).

The *IL6* mRNA expression was significantly elevated 2.5-fold in si*XYLT2*-M0 macrophages and 3.7-fold in si*XYLT2*-M2 macrophages, whereas M1 macrophages showed a significant decrease of 41% following *XYLT2* knockdown (**Figure 2B**). These findings were corroborated at the protein level: IL6 concentrations in cell culture supernatants were significantly reduced 56% in si*XYLT2*-M1 macrophages compared to siNC controls, with a significant elevation of 3.5-fold in si*XYLT2*-M0 and 3.3-fold in si*XYLT2-*M2 macrophages (**Figure 2C**).

Analysis of M2-associated marker genes revealed that *CD163* mRNA expression was significantly induced 5.5-fold in si*XYLT2*-M1 macrophages, while it was significantly reduced 37% in si*XYLT2*-M2 macrophages, and no change was detected in si*XYLT2-*M0 (**Figure 2D**). Similarly, *CD206* mRNA expression was significantly downregulated by 32% and 35% in both M0 and M2 macrophages, respectively, following *XYLT2* knockdown, while it was upregulated 3-fold in M1 macrophages (**Figure 2E**). At the protein level, CD206 surface expression assessed by flow cytometry showed a significant reduction of about 32% in si*XYLT2*-M0 and 39% in *siXYLT2-*M2, while it tended to be elevated in si*XYLT2-*M1 macrophages (**Figure 2F**).

Phagocytic activity was assessed using fluorescently labeled bioparticles to evaluate whether *XYLT2* deficiency also affects the macrophage function. Representative fluorescence microscopy images demonstrate the internalization of phagocytic particles in all conditions (**Figure 2G**). Quantitative image analysis revealed a lower phagocytic activity in siNC-M1 than in siNC-M0 and siNC-M2 macrophages. There was a significant 1.9-fold increase in phagocytic activity in si*XYLT2*-M1 macrophages after the knockdown compared to siNC controls, and a significant reduction of about 25% in si*XYLT2*-M2 macrophages. No difference was observed in M0 macrophages (**Figure 2H**).

**Figure 3:**
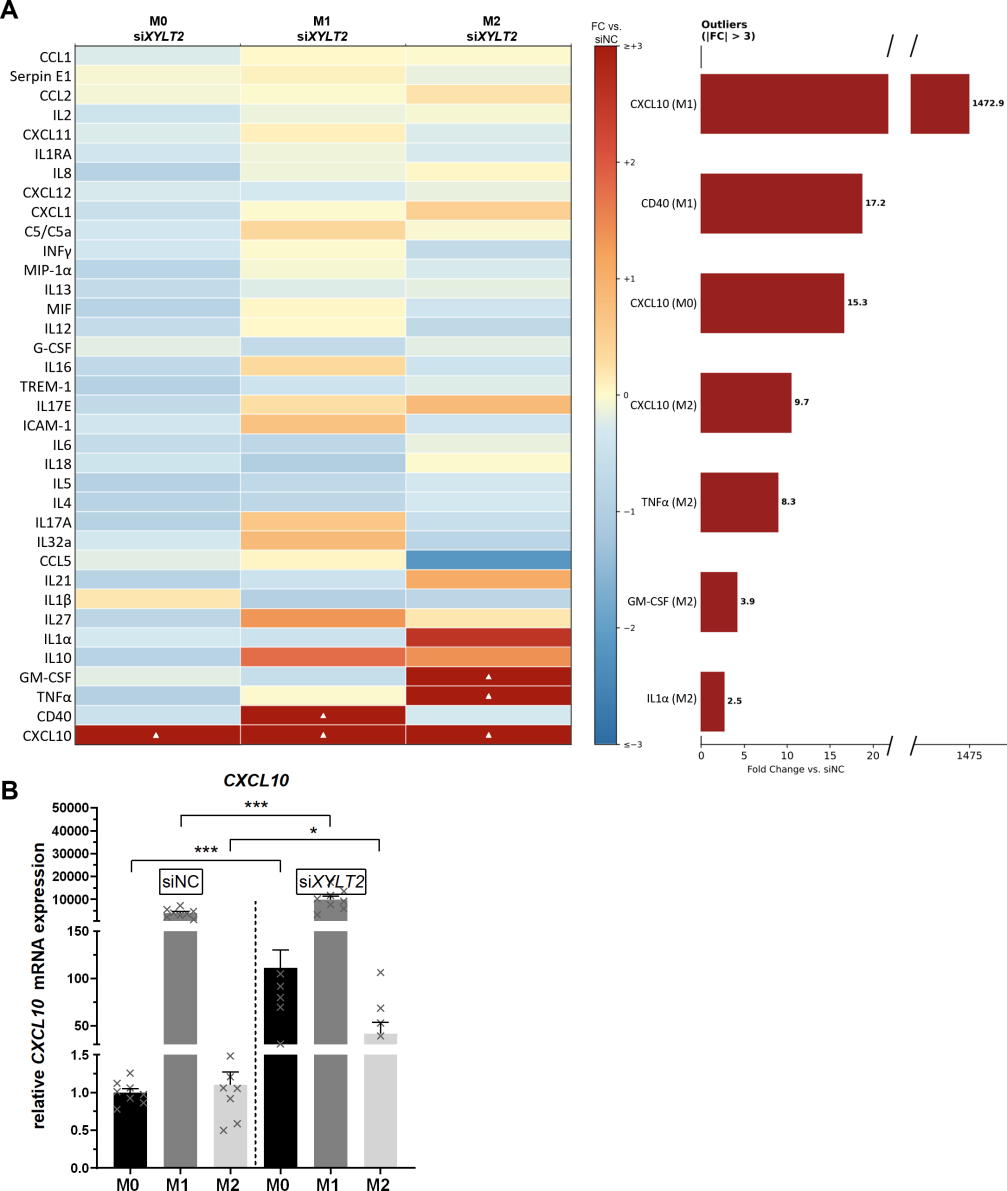
Cytokine secretion profile of polarized macrophages after siRNA-mediated XYLT2 knockdown. Negatively selected monocytes were differentiated into macrophages using M-CSF. Macrophages were treated with a non-targeting negative control siRNA (siNC) or a siRNA targeting XYLT2 (siXYLT2) on day 5. On day 6, macrophages were stimulated with IFNγ/LPS (M1), IL4 (M2), or with no additive (M0). Cells were harvested for qRT-PCR analysis (n =8) or cell culture supernatants were collected after 48 h of polarization and analyzed using a human cytokine array (pooled supernatants, n = 8; 1 biological and 1 technical replicates per condition). **(A)** Heatmap showing relative cytokine secretion levels expressed as fold change (FC) of siXYLT2 versus siNC controls across macrophage polarization states (M0, M1, M2). Cytokines exceeding a FC threshold of 3 were classified as outliers and are summarized in the adjacent bar plot. **(B)** CXCL10 expression measured by qRT-PCR (n = 8; 2 biological and 3 technical replicates per condition). Data are shown as mean ± SEM. Statistical significance was determined using Two-Way-ANOVA: p ≤ 0.05 (*), p ≤ 0.001 (***).

Cell culture supernatants were analyzed using a human cytokine array to obtain a broader cytokine secretion profile. Relative signal intensities of si*XYLT2*-macrophages were expressed as FC versus siNC controls. The heatmap overview revealed differential changes in cytokine secretion across macrophage polarization states following *XYLT2* knockdown (**Figure 3A**). While si*XYLT2-*M0 macrophages showed predominantly reduced cytokine levels, one outlier was detected with CXCL10 (FC 15.3). More pronounced alterations were observed in polarized macrophages. The si*XYLT2-*M2 macrophages displayed a clear increase in several pro-inflammatory mediators, although si*XYLT2-*M1 macrophages exhibited a more selective cytokine response. Several cytokines exceeded an FC threshold of 3 and were classified as outliers. The strongest induction was detected for CXCL10 in *siXYLT2-*M1 macrophages, with an FC of 1472.9 compared to siNC controls. Additional outliers included CD40 in *siXYLT2-*M1 macrophages (FC 17.2), CXCL10 in *siXYLT2-*M2 macrophages (FC 9.7), TNFα in *siXYLT2-*M2 macrophages (FC 6.3), GM-CSF in *siXYLT2-*M2 macrophages (FC 3.9), and IL1α in *siXYLT2-*M2 macrophages (FC 2.5). Given the exceptional upregulation of CXCL10, its mRNA expression was further analyzed by qRT-PCR. The *CXCL10* mRNA analysis revealed a higher expression in siNC-M1 than in siNC-M0 and siNC-M2 macrophages. Furthermore, the *CXCL10* expression was significantly elevated 2.5-fold in si*XYLT2*-M1 macrophages compared to siNC controls, and a significant upregulation about 108-fold and 41-fold was also observed in si*XYLT2*-M0 and si*XYLT2*-M2 macrophages, respectively (**Figure 3B**).

Together, these findings demonstrate that *XYLT2* deficiency disrupts macrophage polarization in a phenotype-specific manner. The *XYLT2* knockdown in M0 macrophages induces features of pro-inflammatory activation, whereas key pro-inflammatory markers are attenuated and functional properties, such as phagocytic activity, are enhanced in M1 macrophages. By contrast, M2 macrophages exhibit a loss of characteristic anti-inflammatory features accompanied by reduced phagocytic activity. These alterations are supported by a broad remodeling of cytokine secretion across all polarization states, most notably characterized by a consistent and pronounced upregulation of CXCL10. Collectively, this indicates a destabilization of macrophage identity rather than a shift toward a defined polarization state. Given the important role of NF-κB in regulating pro-inflammatory macrophage responses, its activity was investigated in the following section.

### *XYLT2* knockdown suppresses NF-κB signaling while promoting STAT1 and STAT3 activation

**Figure 4:**
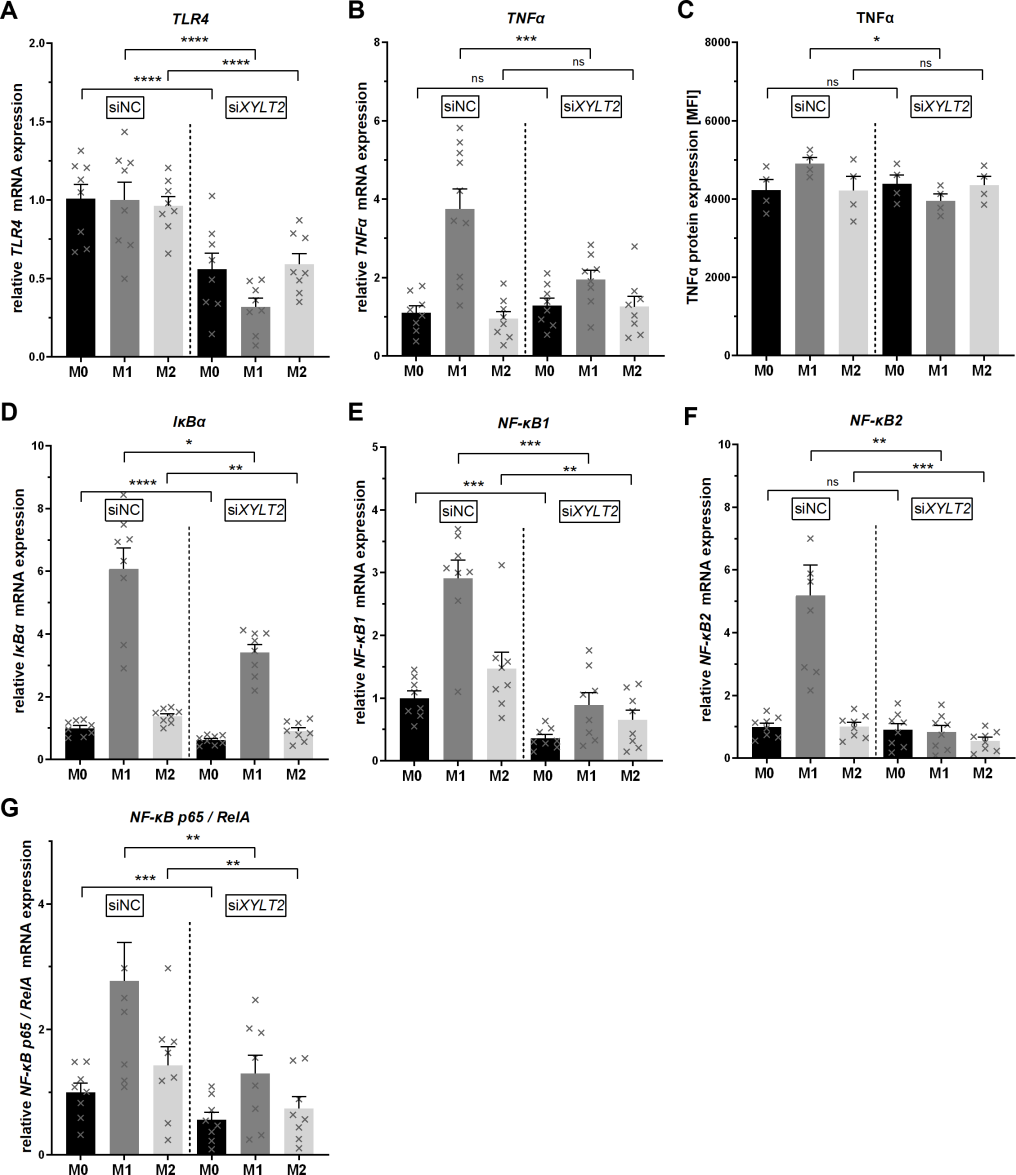
Expression analysis of components of NF-κB signaling pathway of polarized macrophages after siRNA-mediated XYLT2 knockdown. Negatively selected monocytes were differentiated into macrophages using M-CSF. Macrophages were treated with a non-targeting negative control siRNA (siNC) or a siRNA targeting XYLT2 (siXYLT2) on day 5. On day 6, macrophages were stimulated with IFNγ/LPS (M1), IL4 (M2), or with no additive (M0). Cells were harvested for qRT-PCR analysis (n = 8; 2 biological and 3 technical replicates per condition)or protein expression (n = 4; 2 biological and 1 technical replicates per condition) after a polarization time of 48 h. The relative gene or protein expression levels of TLR4 **(A)**, TNFα **(B)**, TNFα **(C)**, IκBα **(D)**, NF-κB1 **(E)**, NF-κB2 **(F)**, and NF-κB p65/RelA **(G)** were analyzed. Data are shown as mean ± SEM. Statistical significance was determined using Two-Way-ANOVA: ns = not significant, p ≤ 0.05 (*), p ≤ 0.01 (**), p ≤ 0.001 (***), p ≤ 0.0001 (****).

The expression of key pathway components was analyzed by qRT-PCR to investigate the impact of *XYLT2* knockdown on the NF-κB signaling pathway (**Figure 4**).

The *TLR4* mRNA expression was significantly reduced in si*XYLT2*-treated macrophages compared to siNC controls in all polarization states, with the most pronounced reduction observed in M1 macrophages (M0: 45%; M1: 68%; M2: 42%) (**Figure 4A**). The *TNFα* mRNA expression was significantly reduced by 51% in si*XYLT2*-M1 macrophages compared to siNC controls, while no significant changes were detected in si*XYLT2-*M0 or si*XYLT2-*M2 macrophages (**Figure 4B**). Intracellular TNFα expression at the protein level, assessed by flow cytometry, was also significantly reduced by 20% in si*XYLT2*-M1 macrophages, with no significant differences observed in either si*XYLT2-*M0 or si*XYLT2-*M2 macrophages (**Figure 4C**). The *IκBα* mRNA expression was significantly reduced in si*XYLT2*-treated macrophages across all polarization states compared to siNC controls: 38% for si*XYLT2-*M0, 42% for si*XYLT2-*M1, and 35% for si*XYLT2-*M2 (**Figure 4D**). This reduction was notably accompanied by a parallel downregulation of the NF-κB family members themselves. The *NF-κB1* (p50) mRNA was significantly downregulated by 6, 64, and 51% in si*XYLT2*-M0, si*XYLT2-*M1, and *siXYLT2-*M2 macrophages, respectively, compared to siNC controls (**Figure 4E**). Similarly, *NF-κB2* (p52) mRNA expression was significantly reduced in all si*XYLT2*-macrophage phenotypes relative to siNC-macrophages (M0: 10%; M1: 84%; M2: 46%) (**Figure 4F**). The *NF-κB p65/RelA* mRNA expression followed the same pattern, with significant reductions of 45, 53, and 48% in si*XYLT2*-M0, si*XYLT2-*M1, and *siXYLT2-*M2 macrophages, respectively (**Figure 4G**).

**Figure 5:**
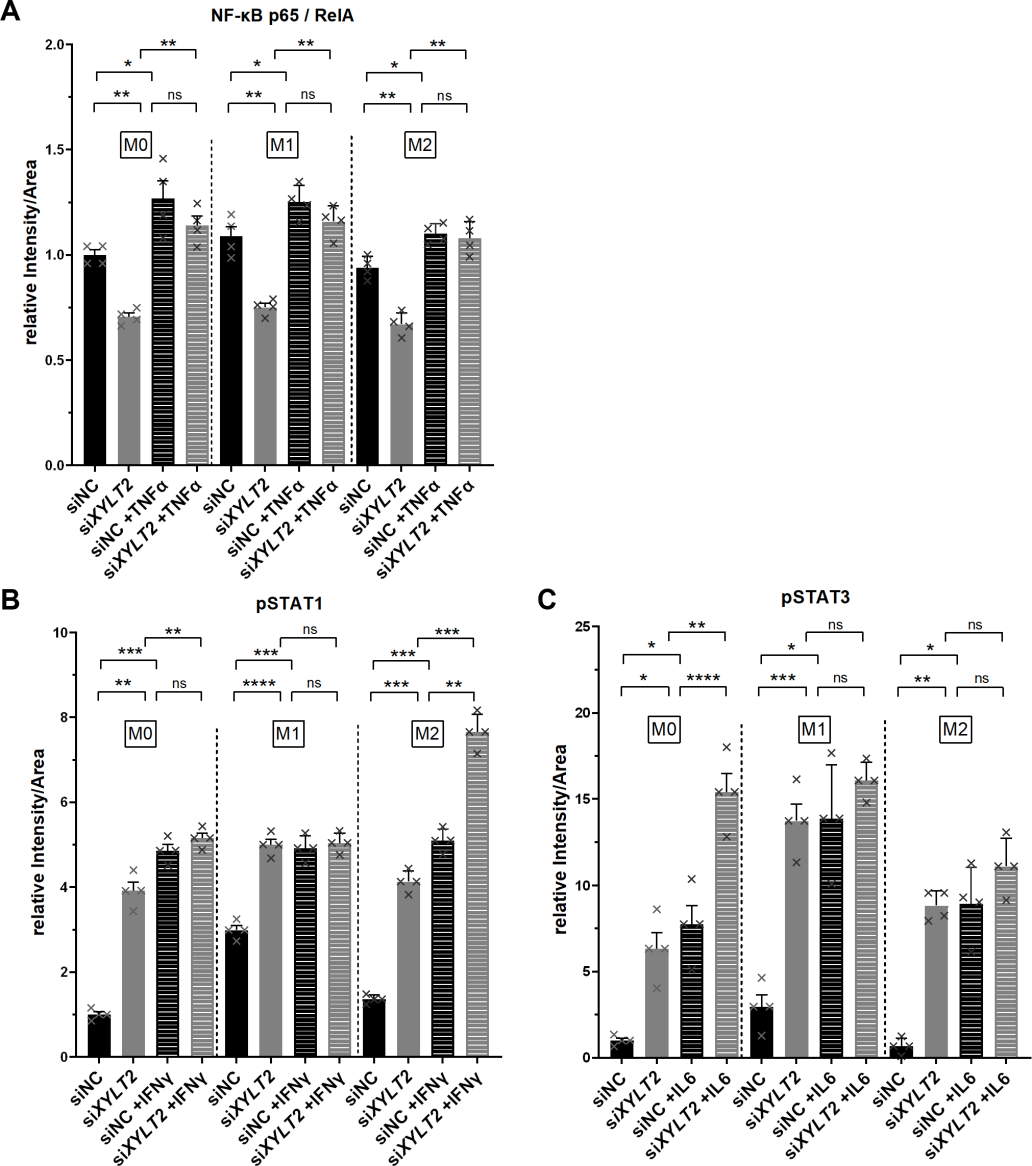
Quantification of nuclear translocation of NF-κB p65, pSTAT1, and pSTAT3 via immunofluorescence analysis in polarized macrophages after siRNA-mediated XYLT2 knockdown. Negatively selected monocytes were differentiated into macrophages using M-CSF. Macrophages were treated with a non-targeting negative control siRNA (siNC) or a siRNA targeting XYLT2 (siXYLT2) on day 5. On day 6, macrophages were stimulated with IFNγ/LPS (M1), IL4 (M2), or without additional stimuli (M0) and polarized for 48 h. Where indicated, macrophages were additionally stimulated with TNFα (20 ng/mL), IFNγ (50 ng/mL), or IL6 (50 ng/mL) for 30 min prior to fixation to assess stimulus-induced signaling responses. Nuclear fluorescence intensities of NF-κB p65/RelA **(A)**, phospho-STAT1 **(B)**, and phospho-STAT3 **(C)** were quantified using ImageJ and normalized to nuclear area (n = 4; 2 biological and 1 technical replicates per condition). Representative immunofluorescence images are shown in **Supplementary Figure 2**. Data are shown as mean ± SEM. Statistical significance was determined using the Two-Way-ANOVA: ns = not significant, p ≤ 0.05 (*), p ≤ 0.01 (**), p ≤ 0.001 (***), p ≤ 0.0001 (****).

The nuclear translocation of NF-κB p65/RelA, pSTAT1, and pSTAT3 was quantified by immunofluorescence microscopy under basal conditions and following pathway-specific stimulation to assess the functional consequences of *XYLT2* knockdown on key inflammatory signaling pathways (**Figure 5**). Representative images are shown in **Supplementary Figure 2**. The nuclear NF-κB p65 intensity was significantly reduced in si*XYLT2* macrophages relative to siNC controls under basal conditions across all phenotypes (M0: 30%; M1: 30%; M2: 23%) (**Figure 5A**). The TNFα stimulation increased the nuclear p65 levels in both siNC and si*XYLT2* conditions; however, the difference between siNC +TNFα and si*XYLT2* +TNFα was nonsignificant across all phenotypes, suggesting that exogenous TNFα stimulation can partially compensate for the *XYLT2* knockdown-associated reduction in p65 activity. By contrast, pSTAT1 nuclear intensity was significantly elevated in si*XYLT2* macrophages compared to siNC controls under basal conditions in all three polarization states: 3.9-fold in si*XYLT2*-M0, 1.7-fold in si*XYLT2*-M1, and 3-fold in si*XYLT2*-M2 (**Figure 5B**). The IFNγ stimulation further increased pSTAT1 levels in both conditions; however, the difference between siNC +IFNγ and si*XYLT2* +IFNγ was nonsignificant in M0 and M1 macrophages, while a significant further elevation by 1.5-fold was observed in si*XYLT2*-M2 macrophages, indicating heightened IFNγ responsiveness in this phenotype following *XYLT2* knockdown. Similarly, phospho-STAT3 nuclear intensity was significantly increased in si*XYLT2*-treated macrophages relative to siNC controls under basal conditions across all polarization states (M0: 6.3-fold; M1: 4.6-fold; M2: 10-fold) (**Figure 5C**). The IL6 stimulation further elevated pSTAT3 levels in both conditions; the difference between siNC +IL6 and si*XYLT2* +IL6 was nonsignificant in M1 and M2 macrophages, while a significant additional 2-fold increase was observed in si*XYLT2*-M0 macrophages. Notably, basal pSTAT3 levels in si*XYLT2* macrophages already approached the magnitude of IL6-induced STAT3 activation observed in siNC controls, suggesting near-maximal constitutive STAT3 activation in this condition.

Taken together, these findings show that *XYLT2* deficiency leads to a consistent shift in intracellular inflammatory signaling across all macrophage polarization states. The NF-κB activity is reduced in M0, M1, and M2 macrophages, which is reflected by the decreased expression of key pathway components and reduced nuclear translocation of p65. By contrast, STAT1 and STAT3 signaling is increased across all phenotypes. While TNFα stimulation largely restores p65 nuclear translocation to comparable levels in siNC and si*XYLT2* macrophages, stimulation-dependent differences in STAT signaling are phenotype-specific, with enhanced IFNγ responsiveness in M2 macrophages and increased IL6 responsiveness in M0 macrophages. This shift notably occurs despite the pro-inflammatory cytokine profile observed previously, indicating that pro-inflammatory activation in *XYLT2*-deficient macrophages is here not driven by canonical NF-κB signaling but, instead, associated with alternative pathways, consistent with sustained STAT1/STAT3 activation. Overall, these results suggest a reprogramming of inflammatory signaling rather than a uniform shift in pro-inflammatory activity in *XYLT2*-deficient macrophages. Transcriptome-wide mRNA sequencing was performed to further evaluate the transcriptional consequences of this signaling shift.

### Transcriptome-wide profiling reveals a conserved rewiring of innate immune signaling upon *XYLT2* deficiency

**Figure 6:**
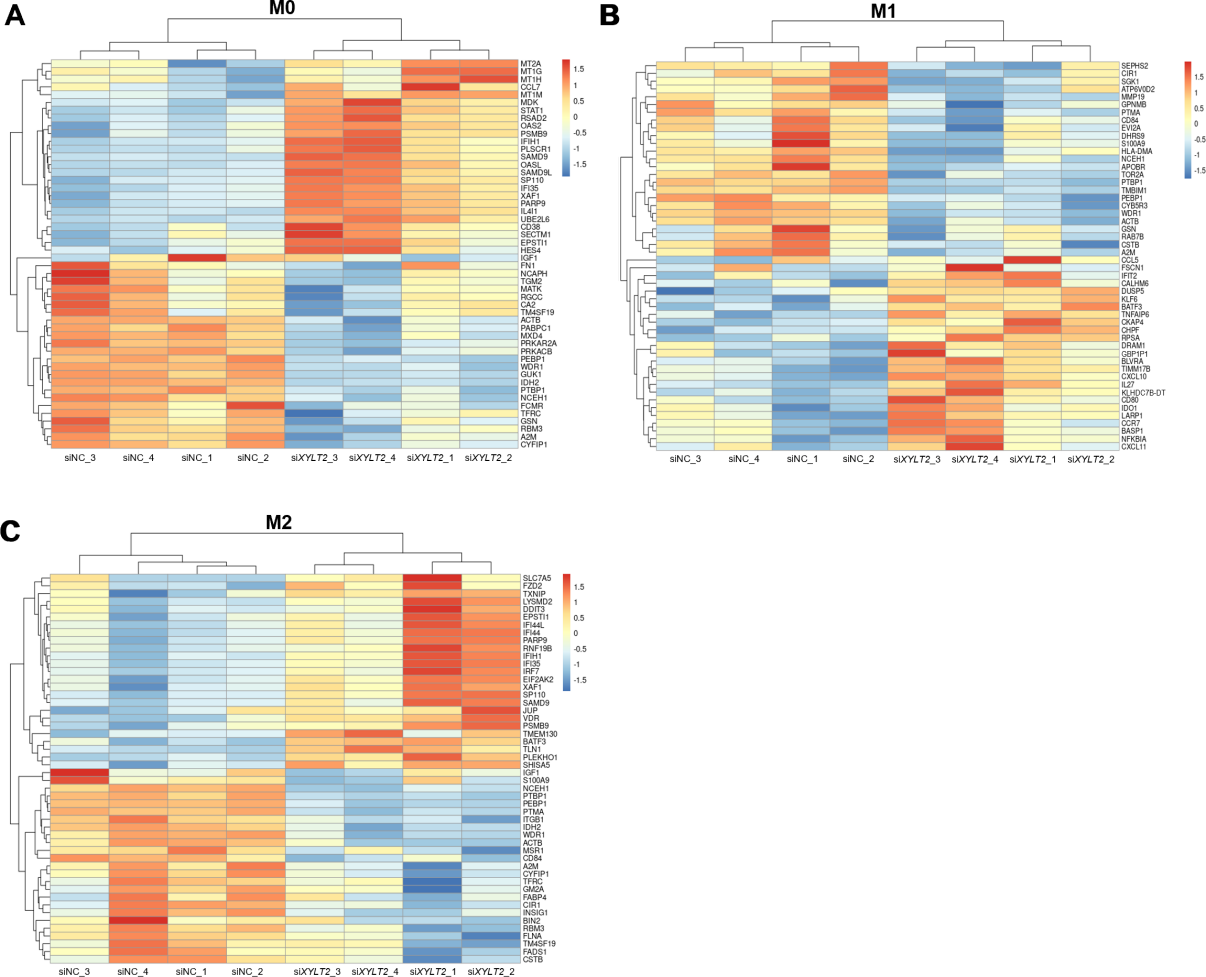
Heatmap visualization of differentially expressed genes in polarized macrophages after siRNA-mediated XYLT2 knockdown. Negatively selected monocytes were differentiated into macrophages using M-CSF. Macrophages were treated with a non-targeting negative control siRNA (siNC) or a siRNA targeting XYLT2 (siXYLT2) on day 5. On day 6, macrophages were stimulated with IFNγ/LPS (M1), IL4 (M2), or with no additive (M0). Cells were harvested for mRNA-Seq (n = 4; 1 biological and 1 technical replicates per condition) after a polarization time of 48 h. The top 25 up- and downregulated DEGs (padj < 0.05, |LFC| ≥ 0.5) are shown for M0 **(A)**, M1 **(B)**, and M2 **(C)** macrophages. Z-score normalized variance stabilizing - transformed counts are displayed. Hierarchical clustering was performed on rows and columns. Red indicates higher expression and blue indicates lower expression relative to the mean expression level of each gene.

Bulk mRNA sequencing was performed in M0, M1, and M2 macrophages following siRNA-mediated *XYLT2* knockdown to characterize the transcriptional consequences of *XYLT2* knockdown at a genome-wide level. Principal component analysis confirmed a consistent separation between siNC and si*XYLT2* conditions across all three polarization states, with donor effects accounted for by the experimental design (**Supplementary Figure 3 A1–A3**). Dispersion estimates fitted well across all conditions, indicating robust model performance (**Supplementary Figure 3 B1–B3**). Both MA and volcano plots confirmed the distribution of DEGs and directionality of FCs (**Supplementary Figure 4**). Hierarchical clustering and heatmap visualization of the top 50 DEGs revealed distinct transcriptional signatures in each polarization state following *XYLT2* knockdown compared to siNC (**Figures 6A–C**).

Upregulated genes in si*XYLT2*-M0 macrophages included ISGs, such as *STAT1*, *OAS2*, *RSAD2*, *IFIT1H1*, *SAMD9*, *SAMD9L*, and *SP110*, alongside *CCL7*, *MDK*, and metallothionein family members (*MT2A*, *MT1G*, *MT1H*, *MT1M*) (**Figure 6A**). Downregulated genes in *siXYLT2-*M0 included structural and cytoskeletal factors, such as *A2M*, *GSN*, *ACTB*, *RBM3*, and *CYFIP1*, as well as *PTBP1*, *PEBP1*, *IGF1*, and *FN1*. Upregulated genes in si*XYLT2*-M1 macrophages included IFN- and immune-associated factors, such as *CXCL10*, *CXCL11*, *IFIT2*, *BATF3*, *S100A9*, *IDO1*, and *IL27*, alongside *CSTB*, *FSCN1*, and *TNFAIP6* (**Figure 6B**). Downregulated genes in si*XYLT2-*M1 included *PTBP1*, *ACTB*, *GSN*, *A2M*, *PEBP1*, *WDR1*, and *RAB7B*. Upregulated genes in si*XYLT2-*M2 macrophages were dominated by ISGs, including *IFI44*, *IFI44L*, *IFIT1H1*, *IRF7*, *EIF2AK2*, *RNF19B*, *SAMD9*, and *EPSTI1*, as well as *BATF3*, *XAF1*, *TXNIP*, and *SLC7A5* (**Figure 6C**). Downregulated genes in si*XYLT2-*M2 included *PTBP1*, *A2M*, *GSN*, *ACTB*, *CIR1*, *FADS1*, *RBM3*, *FLNA*, *TM4SF19*, and *CSTB*.

**Figure 7:**
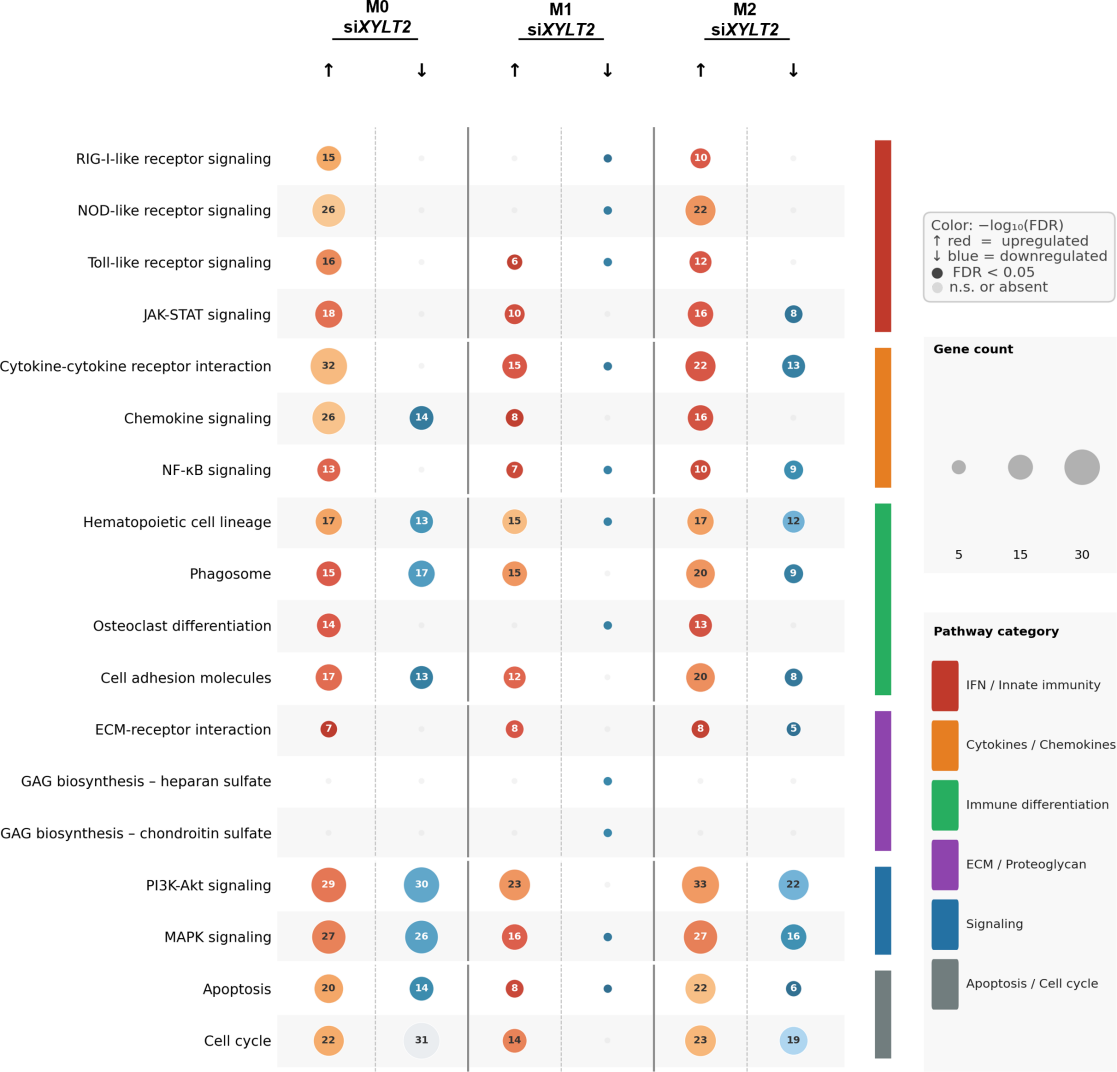
KEGG pathway enrichment analysis of DEGs in polarized macrophages after siRNA-mediated XYLT2 knockdown. Negatively selected monocytes were differentiated into macrophages using M-CSF. Macrophages were treated with a non-targeting negative control siRNA (siNC) or a siRNA targeting XYLT2 (siXYLT2) on day 5. On day 6, macrophages were stimulated with IFNγ/LPS (M1), IL4 (M2), or with no additive (M0). Cells were harvested for mRNA-Seq (n = 4; 1 biological and 1 technical replicates per condition) after a polarization time of 48 h. Dot plot representation of significantly enriched pathways in M0, M1, and M2 macrophages following XYLT2 knockdown is shown. Pathways are grouped according to functional categories, with upregulated (↑) and downregulated (↓) DEGs. Circle size indicates the number of DEGs associated with each pathway, while color intensity represents the statistical significance (−log10 false discovery rate). Red circles indicate pathways enriched in upregulated genes, whereas blue circles represent pathways enriched in downregulated genes, and color intensity reflects the −log10 false discovery rate. Only pathways with a false discovery rate < 0.05 are shown. n.s., not significant or absent. mediated XYLT2 knockdown. Negatively selected monocytes were differentiated into macrophages using M-CSF. On day 5, macrophages were treated with a non-targeting negative control siRNA (siNC) or a siRNA targeting XYLT2 (siXYLT2). On day 6, macrophages were stimulated with IFNγ/LPS (M1), IL4 (M2) or with no additive (M0). Cells were harvested for mRNA-Seq (ndonor= 4; nbiological = 1; ntechnical = 1) after a polarization time of 48 h. Dot plot representation of significantly enriched pathways in M0, M1, and M2 macrophages following XYLT2 knockdown is shown. Pathways are grouped according to functional categories, with upregulated (↑) and downregulated (↓) DEGs. Circle size indicates the number of differentially expressed genes associated with each pathway, while color intensity represents the statistical significance (−log10 FDR). Red circles indicate pathways enriched in upregulated genes, whereas blue circles represent pathways enriched in downregulated genes, color intensity reflects the −log10(FDR). Only pathways with FDR < 0.05 are shown. n.s.: not significant or absent.

Pathway enrichment analysis revealed a consistent pattern of transcriptional rewiring across all three polarization states after *XYLT2* knockdown (**Figure 7**). Upregulated DEGs were significantly enriched in innate immune signaling pathways, including RIG-I-like receptor signaling, NOD-like receptor signaling, Toll-like receptor signaling, and JAK-STAT signaling across si*XYLT2*-M0 and si*XYLT2*-M2 macrophages. Cytokine-cytokine receptor interaction and chemokine signaling pathways were also significantly enriched among upregulated genes in all phenotypes. The NF-κB signaling showed enrichment among not only upregulated genes in si*XYLT2*-M0 (13 genes), si*XYLT2*-M1 (7 genes), and si*XYLT2*-M2 (10 genes), but also downregulated genes in si*XYLT2*-M2 (9 genes), reflecting the complex bidirectional dysregulation of this pathway. Enrichment of phagosome-associated genes among upregulated DEGs in si*XYLT2*-M0 (15 genes) and si*XYLT2*-M2 (20 genes) macrophages provides transcriptional corroboration for the altered phagocytic activity observed in the functional assays, suggesting that *XYLT2* deficiency affects not only phagocytic activity but also the broader transcriptional program governing phagosomal biology. Osteoclast differentiation was significantly enriched among upregulated DEGs in si*XYLT2*-M0 (14 genes) and si*XYLT2*-M2 (13 genes) macrophages, but not in si*XYLT2*-M1 macrophages. Given that macrophages belong to the monocyte-macrophage-osteoclast axis and that M2 macrophages physiologically suppress osteoclast differentiation, the upregulation of osteoclast differentiation-associated genes in si*XYLT2*-M2 macrophages is particularly noteworthy. This finding suggests that *XYLT2* deficiency may shift si*XYLT2*-M0 and si*XYLT2*-M2 macrophages toward a pro-osteoclastogenic transcriptional state. The ECM receptor interaction pathways were also enriched among upregulated DEGs across multiple phenotypes (M0: 7 genes; M1: 8 genes; M2: 8 genes), consistent with the known role of PGs in mediating ECM cell signaling and further linking the loss of XT-II enzymatic activity to broader ECM dysregulation. Pathways related to GAG biosynthesis – specifically heparan sulfate and chondroitin sulfate biosynthesis – were significantly enriched among downregulated genes exclusively in si*XYLT2*-M1 macrophages, with no significant enrichment detected in si*XYLT2*-M0 or si*XYLT2*-M2 macrophages. This phenotype-specific finding directly reflects the primary enzymatic function of XT-II and confirms that *XYLT2* knockdown exerts functionally relevant transcriptional effects on PG biosynthetic genes, particularly in the context of M1 polarization. Both PI3K-Akt and MAPK signaling showed the most complex enrichment patterns, with both up- and downregulated gene sets significantly enriched across all three polarization states. The PI3K-Akt signaling in si*XYLT2*-M0 macrophages was enriched among both upregulated (29 genes) and downregulated (30 genes) DEGs, indicating broad but opposing transcriptional changes within this pathway. Similarly, MAPK signaling was enriched among upregulated (27 genes) and downregulated (26 genes) genes in si*XYLT2*-M0, and among upregulated (27 genes) and downregulated (16 genes) genes in si*XYLT2*-M2 macrophages. This bidirectional enrichment pattern suggests a fundamental disruption of intracellular signal transduction rather than a uniform activation or suppression, the consequences of which for macrophage function warrant further investigation.

Collectively, these findings demonstrate that *XYLT2* deficiency induces a pronounced and conserved IFN-associated transcriptional program across all macrophage polarization states, as reflected by the consistent upregulation of ISGs. This is accompanied by coordinated changes in innate immune and cytokine signaling pathways, including JAK-STAT and pattern recognition receptor signaling, while other pathways, such as NF-κB, PI3K-Akt, and MAPK, display more complex, bidirectional regulation. Overall, these results reinforce the hypothesis of a global reprogramming of inflammatory signaling toward an IFN-dominated state induced by *XYLT2* deficiency.

## Discussion

Pathogen mutations in *XYLT2* cause SOS, which manifests as severe primary osteoporosis and skeletal abnormalities, along with cataract, hearing impairments, and effects on the cardiovascular system (37). Dysregulated macrophage polarization has recently been closely linked to bone remodeling disorders. Macrophages are not only considered precursors of bone-resorbing osteoclasts but also actively regulate their differentiation. M1 macrophages secrete pro-inflammatory factors, such as IL1β, to mature osteoclast precursors, whereas M2 macrophages inhibit osteoclast differentiation from precursor cells by secreting IL4 and IL10 (30, 52, 53). Consequently, a persistent shift toward M1-dominated inflammatory states can drive excessive osteoclast activity and contribute to the development of osteoporosis (54). Previous work from our group identified the XT isoform XT-I, encoded by *XYLT1*, as a polarization-responsive gene that is upregulated in M2 macrophages. However, siRNA-mediated knockdown of *XYLT1* did not alter the macrophage polarization, suggesting that XT-I is not essential for maintaining macrophage activation programs (43). By contrast, the functional role of XT-II, encoded by *XYLT2*, in macrophages remains largely unknown. Given the important role of macrophages in regulating osteoclast differentiation and bone homeostasis, alterations in XT-II activity may have direct consequences for osteoclastogenesis and thereby contribute to bone-related pathologies, such as the osteoporosis characteristic of SOS. We, therefore, investigated in the present study whether XT-II deficiency affects macrophage polarization and inflammatory signaling in human primary macrophages, and if its loss may represent a previously unrecognized pathomechanism contributing to SOS through the dysregulation of immune cell function.

The siRNA-mediated knockdown of *XYLT2* resulted in a significant reduction of both XT-II and XT-I activity across all macrophage phenotypes, indicating a functional coupling between the two XT isoforms. Considering this, it should be mentioned that *XYLT1* knockdown does not affect *XYLT2* expression in macrophages, as previously demonstrated by Ly et al. (43). In order to confirm the specificity of this finding and exclude methodological off-target effects, *XYLT2* knockdown was additionally performed using another *XYLT2*-targeting siRNA (ID 112168, Thermo Fisher Scientific, Waltham, MA, USA); the resulting data were consistent with the findings presented here, supporting the validity of the method (data not shown).The co-reduction of XT-I activity following the *XYLT2* knockdown observed suggests that XT-II may act as the dominant regulator of overall XT capacity in human primary macrophages, consistent with its ubiquitous expression pattern and role as the predominant isoform in most cell types (39, 55). In support of this, Kleine et al. have already shown that a *XYLT2* knockdown in human dermal fibroblasts can lead to a concurrent reduction in *XYLT1* expression and XT-I activity (56). The molecular basis of this coupling remains unclear but may involve shared transcriptional regulation or feedback mechanisms within the PG biosynthetic pathway. Furthermore, *XYLT2* mRNA expression was elevated in M2 macrophages under control conditions, mirroring the polarization-dependent upregulation of *XYLT1*. This parallel suggests that both XT isoforms may be transcriptionally responsive to the M2 polarization program, potentially reflecting an increased demand for PG biosynthesis in anti-inflammatory macrophages. Indeed, M2 macrophages are characterized by enhanced ECM remodeling activity and elevated expression of heparan sulfate PG biosynthesis enzymes compared to M1 macrophages (20).

In addition to its consequences on XT-I, *XYLT2* knockdown profoundly altered the macrophage phenotype and function. Analysis of classical polarization markers revealed a destabilization of polarization programs across all macrophage phenotypes, resulting in a partial convergence of M1- and M2-associated features. The *XYLT2* deficiency in unpolarized si*XYLT2*-M0 macrophages was sufficient to drive a pro-inflammatory shift, as evidenced by the increased expression of pro-inflammatory cytokines, downregulation of the M2-associated marker CD206, and acquisition of a rounded morphology reminiscent of M1 activation (56), suggesting that XT-II contributes to the maintenance of macrophage homeostasis in the resting state. The *XYLT2* deficiency in functionally polarized macrophages resulted in a destabilization of established activation programs: si*XYLT2*-M2 macrophages showed a reduced expression of M2-associated markers, including CD206, alongside increased pro-inflammatory cytokine expression and a tendency toward a more rounded morphology, indicating a partial loss of M2 identity. Conversely, si*XYLT2*-M1 macrophages exhibited a reduced expression of classical pro-inflammatory markers combined with a partial induction of M2-associated features, but no morphological changes were observed, suggesting that the established M1 program is more resistant to the consequences of *XYLT2* deficiency. Together, these findings indicate that XT-II is not required to initiate macrophage polarization, but rather contributes to the stabilization of distinct activation states, with resting and anti-inflammatory macrophages showing the highest susceptibility to its loss. The functional consequences of this destabilization were evident in altered phagocytic activity. CD206, a mannose receptor critically involved in endocytosis and phagocytosis (57), was significantly downregulated in si*XYLT2*-M2 macrophages and correspondingly associated with reduced phagocytic capacity. By contrast, increased CD206 expression in si*XYLT2*-M1 macrophages was accompanied by enhanced phagocytic activity. These findings suggest that *XYLT2* deficiency alters phagocytosis in a receptor-dependent manner and further supports the notion of functional reprogramming rather than a simple shift in polarization.

Analysis of the cytokine secretion profile revealed a polarization-dependent dysregulation of inflammatory mediators following *XYLT2* knockdown. Cytokine levels in si*XYLT2*-M0 macrophages were slightly reduced, consistent with the low basal secretory activity of unpolarized macrophages (58), suggesting that transcriptional changes are not immediately translated into altered protein secretion. By contrast, polarized macrophages exhibited more pronounced alterations, with si*XYLT2*-M2 macrophages showing an increased secretion of pro-inflammatory mediators and si*XYLT2*-M1 macrophages displaying a more selective response. Notably, downregulated cytokines were largely absent, indicating that *XYLT2* deficiency predominantly promotes rather than suppresses inflammatory secretion in activated macrophages. A particular finding was the consistent upregulation of CXCL10 across all macrophage polarization states. CXCL10, also known as IFNγ -induced protein 10, is a chemokine canonically induced by IFNγ through the JAK-STAT1 and interferon regulatory factor signaling axes and serves as a hallmark target gene of ISG expression programs (59, 60). Its strong induction in si*XYLT2*-M1 macrophages suggests the synergistic amplification of IFN signaling, while its upregulation in si*XYLT2*-M0 and si*XYLT2*-M2 macrophages in the absence of exogenous IFNγ indicates the constitutive activation of IFN-associated pathways (61). Additional induction of cytokines, such as CD40, TNFα, GM-CSF, and IL1α, further supports a shift toward a pro-inflammatory secretory profile (14).

The NF-κB signaling pathway is responsible for the synthesis of many pro-inflammatory cytokines and the important transcriptional regulator of pro-inflammatory macrophage activation (8), and was, therefore, examined in greater detail. The *XYLT2* knockdown resulted in a coordinated downregulation of multiple NF-κB pathway components at the transcriptional level, accompanied by the reduced nuclear translocation of p65 across all polarization states, collectively indicating an impairment of canonical NF-κB signaling activity. This could be a possible explanation for the downregulation of pro-inflammatory markers in si*XYLT2*-M1 macrophages. Reduced TLR4 expression provides a plausible upstream mechanism, as TLR4 mediates LPS-induced NF-κB activation (62). In addition, heparan sulfate and small leucine-rich PGs act as co-receptors facilitating TLR4 ligand presentation and receptor clustering, and their loss may further impair signal transduction (63–65). The concurrent downregulation of *NF-κB1*, *NF-κB2*, and *NF-κB p65/RelA* mRNA suggests that *XYLT2* deficiency suppresses the NF-κB pathway not only through reduced upstream receptor signaling but also by limiting the availability of the transcription factor subunits required for dimer formation and target gene expression (66). The reduction in *IκBα* mRNA following *XYLT2* knockdown warrants particular attention, as IκBα is the primary cytoplasmic inhibitor of NF-κB and its expression is itself driven by NF-κB activity as part of a negative feedback loop (67). A reduction in *IκBα* transcription may, therefore, reflect the overall suppression of NF-κB transcriptional activity rather than representing an independent regulatory event. This interpretation is consistent with the reduced nuclear p65 levels observed by immunofluorescence, which confirm that, despite lower IκBα-mediated inhibition, p65 nuclear translocation remains impaired. Importantly, exogenous TNFα stimulation was able to induce p65 nuclear translocation in si*XYLT2* macrophages to levels comparable to those observed in stimulated siNC controls, indicating that the capacity for stimulus-induced NF-κB activation is preserved following *XYLT2* knockdown. This suggests that *XYLT2* deficiency primarily affects the basal set point of NF-κB activity rather than abolishing the pathway’s responsiveness to external stimuli. One explanation for the widespread transcriptional downregulation of NF-κB signaling pathway components is the loss of autoregulatory amplification resulting in a self-reinforcing downward spiral (8, 66).

Crosstalk between NF-κB and STAT1 provides a mechanistic explanation for this signaling imbalance. STAT1, activated by IFN through JAK1/JAK2, can suppress NF-κB signaling by competing for promoter binding and interacting with NF-κB complexes (15, 18, 68, 69). Consequently, a *XYLT2* knockdown leads to a constitutive elevation of pSTAT1 across all macrophage polarization states, even in the absence of exogenous cytokine stimulation, suggesting that *XYLT2* deficiency is sufficient to engage these pathways autonomously. In this view, the reduction in NF-κB component expression and the elevation of STAT1 activity may not represent independent consequences of *XYLT2* deficiency, but rather two facets of a single interconnected signaling imbalance. In addition to STAT1, STAT3 signaling was also markedly affected by *XYLT2* knockdown, resulting in constitutive pSTAT3 activation across all macrophage phenotypes. Regarding STAT3, its functional output is context-dependent: while IL10-driven STAT3 activation in M2 macrophages promotes anti-inflammatory programs and suppresses NF-κB activity, IL6-driven STAT3 activation reinforces pro-inflammatory gene expression (23). The constitutive activation of STAT1 and STAT3 provides a plausible mechanistic explanation for the dysregulation of polarization-associated marker genes observed following *XYLT2* knockdown. STAT1 signaling is known to suppress the expression of M2-associated markers, including CD206 and CD163, by antagonizing the IL4/STAT6 axis that drives their transcription, which would be consistent with the significant downregulation of both markers in si*XYLT2*-M2 macrophages (70, 71). Conversely, the paradoxical induction of CD163 and CD206 in si*XYLT2*-M1 macrophages may reflect the context-dependent interplay between STAT1 and STAT3 in cells which are already activated by IFNγ/LPS, where sustained STAT3 activity has been shown to partially counteract the STAT1-driven suppression of M2 marker expression (72–74). Furthermore, the pro-inflammatory shift in M0 macrophages, including the upregulation of IL1β and IL6, is consistent with the known capacity of constitutively active STAT3 to drive pro-inflammatory cytokine expression in the absence of classical polarizing stimuli (75, 76). The constitutive activation of both STAT1 and STAT3 in the absence of exogenous cytokine stimulation suggests the presence of an endogenous activating signal upstream of the JAK-STAT axis (77). One compelling hypothesis is that fragments of incompletely synthesized or degraded PGs resulting from impaired XT-II activity may accumulate and act as damage-associated molecular patterns, triggering innate immune pattern recognition receptor activation and downstream JAK-STAT1 and JAK-STAT3 signaling (78–80). Additionally, impaired glycosylation of PG core proteins may lead to the accumulation of misfolded proteins in the endoplasmic reticulum (81). Activation of the unfolded protein response under these conditions has been shown to promote pro-inflammatory signaling, and chronic endoplasmic reticulum stress can further amplify innate immune activation through the engagement of pattern recognition receptor pathways (82, 83). Together, these mechanisms would provide a direct mechanistic link between the enzymatic loss of XT-II, PG biosynthesis impairment, and the IFN-like transcriptional response observed, including the exceptional induction of CXCL10 and the broad pro-inflammatory cytokine shift. Bulk mRNA sequencing was performed across all three polarization states to further characterize the transcriptional consequences of *XYLT2* deficiency and identify the signaling pathways underlying this response at a genome-wide level.

Transcriptome-wide profiling by bulk mRNA sequencing confirmed and extended the findings from targeted analyses. Hierarchical clustering revealed distinct but overlapping transcriptional signatures across M0, M1, and M2 macrophages following *XYLT2* knockdown. A shared core of ISGs – including *SAMD9*, *SAMD9L*, *IRF7*, *EIF2AK2*, and *IFI44* family members – in si*XYLT2*-M0 and si*XYLT2*-M2 macrophages was consistently upregulated, consistent with the constitutive pSTAT1 elevation observed by immunofluorescence and providing genome-wide corroboration for the IFN-skewed transcriptional program induced by *XYLT2* deficiency (84–86). The most prominent upregulated genes in si*XYLT2*-M1 macrophages included IFN-associated mediators, such as *CXCL10*, *CXCL11*, *IDO1*, and *BATF3*, reflecting a more selective but equally IFN-dominated transcriptional response (84, 85). Pathway enrichment analysis revealed significant upregulation of RIG-I-, NOD-, and Toll-like receptor signaling pathways across si*XYLT2*-M0 and si*XYLT2*-M2 macrophages, directly supporting the damage-associated molecular pattern hypothesis outlined above (87). The concurrent enrichment of JAK-STAT and cytokine-cytokine receptor interaction pathways across all polarization states corroborates the important role of this signaling axis in mediating the transcriptional consequences of *XYLT2* knockdown. It should be noted, however, that the NF-κB signaling pathways appeared upregulated in the transcriptome-wide analysis across si*XYLT2*-M0, si*XYLT2*-M1 and si*XYLT2*-M2 macrophages, which may appear to contradict the shown suppression of canonical NF-κB activity. However, KEGG pathway enrichment captures all genes annotated to the NF-κB pathway, including upstream innate immune sensing components such as pattern recognition receptors and downstream signal mediators, rather than canonical transcription factor activity per se. This is consistent with the concurrent enrichment of RIG-I-like, NOD-like and Toll-like receptor signaling pathways in the same dataset. The transcriptomic findings therefore do not directly contradict but rather contextualize the functional NF-κB suppression observed at the molecular level and the complex dysregulation of this pathway warrants further investigation. The exclusive downregulation of GAG biosynthesis pathways in si*XYLT2*-M1 macrophages directly confirms the functional impact of *XYLT2* knockdown on PG biosynthetic gene networks (88). Finally, the enrichment of osteoclast differentiation pathways among upregulated DEGs in si*XYLT2*-M0 and si*XYLT2*-M2 macrophages suggests that *XYLT2* deficiency may induce transcriptional programs associated with osteoclast differentiation, providing a potential mechanistic link to the severe osteoporosis observed in SOS patients.

The data obtained in this study demonstrate for the first time that XT-II fulfills a functionally significant role in macrophage polarization and inflammatory signaling in human primary macrophages. The *XYLT2* deficiency induces a coordinated reduction of XT-I and XT-II enzymatic activity, destabilizes M1 and M2 macrophage identity, promotes spontaneous pro-inflammatory activation in resting M0 macrophages, and induces a reciprocal shift between NF-κB suppression and constitutive STAT1 and STAT3 activation, a signaling imbalance that is reflected at the transcriptome-wide level in a conserved IFN-associated gene expression program across all polarization states. These findings extend the known pathophysiological consequences of *XYLT2* deficiency and raise the possibility that dysregulated macrophage immune function represents a previously unrecognized component of SOS. In the context of bone homeostasis, the pro-inflammatory and osteoclast-associated transcriptional changes observed in *XYLT2*-deficient M0 and M2 macrophages may contribute to the severe osteoporosis characteristic of SOS, identifying macrophage polarization as a potential cellular mechanism linking PG biosynthesis defects to skeletal pathology.

## Data Availability

The mRNA sequencing data generated in this study have been deposited in the NCBI Gene Expression Omnibus (GEO) repository and are publicly accessible under accession number GSE329556.

## References

[1] Rieger MA Schroeder T . Hematopoiesis. Cold Spring Harb Perspect Biol. (2012) 4:a008250. doi: 10.1101/cshperspect.a008250 23209149 PMC3504436

[2] Orekhov AN Orekhova VA Nikiforov NG Myasoedova VA Grechko AV Romanenko EB . Monocyte differentiation and macrophage polarization. Vessel Plus. (2019) 3:N/A–A. doi: 10.20517/2574-1209.2019.04

[3] Gordts PLSM Esko JD . Heparan sulfate proteoglycans fine-tune macrophage inflammation via IFN-β. Cytokine. (2015) 72:118–9. doi: 10.1016/j.cyto.2014.12.013 25573804

[4] Lis-López L Bauset C Seco-Cervera M Cosín-Roger J . Is the macrophage phenotype determinant for fibrosis development? Biomedicines. (2021) 9. doi: 10.3390/biomedicines9121747 34944564 PMC8698841

[5] Huang X Li Y Fu M Xin HB . Polarizing macrophages *in vitro*. Methods Mol Biol. (2018) 1784:119–26. doi: 10.1007/978-1-4939-7837-3_12 29761394 PMC8875934

[6] Sica A Mantovani A . Macrophage plasticity and polarization: *in vivo* veritas. J Clin Invest. (2012) 122:787–95. doi: 10.1172/JCI59643 22378047 PMC3287223

[7] Yunna C Mengru H Lei W Weidong C . Macrophage M1/M2 polarization. Eur J Pharmacol. (2020) 877:173090. doi: 10.1016/j.ejphar.2020.173090 32234529

[8] Dorrington MG Fraser IDC . NF-κB signaling in macrophages: dynamics, crosstalk, and signal integration. Front Immunol. (2019) 10:705. doi: 10.3389/fimmu.2019.00705 31024544 PMC6465568

[9] King DJ Bassett SE Li X Fennewald SA Herzog NK Luxon BA . Combinatorial selection and binding of phosphorothioate aptamers targeting human NF-kappa B RelA(p65) and p50. Biochemistry. (2002) 41:9696–706. doi: 10.1021/bi020220k 12135392

[10] Zhang G Ghosh S . Toll-like receptor-mediated NF-kappaB activation: a phylogenetically conserved paradigm in innate immunity. J Clin Invest. (2001) 107:13–9. doi: 10.1172/JCI11837 11134172 PMC198554

[11] Kaltschmidt B Widera D Kaltschmidt C . Signaling via NF-kappaB in the nervous system. Biochim Biophys Acta. (2005) 1745:287–99. doi: 10.1016/j.bbamcr.2005.05.009 15993497

[12] Ward PA Lentsch AB . Endogenous regulation of the acute inflammatory response. Mol Cell Biochem. (2002) 234–235:225–8. doi: 10.1007/978-1-4615-1087-1_26 12162438

[13] Hu X Ivashkiv LB . Cross-regulation of signaling and immune responses by IFN-γ and STAT1. Immunity. (2009) 31:539–50. doi: 10.1016/j.immuni.2009.09.002 19833085 PMC2774226

[14] Delgado M . Inhibition of interferon (IFN) gamma-induced Jak-STAT1 activation in microglia by vasoactive intestinal peptide: inhibitory effect on CD40, IFN-induced protein-10, and inducible nitric-oxide synthase expression. J Biol Chem. (2003) 278:27620–9. doi: 10.1074/jbc.M303199200 12754213

[15] Krämer OH Baus D Knauer SK Stein S Jäger E Stauber RH . Acetylation of Stat1 modulates NF-κB activity. Genes Dev. (2006) 20:473–85. doi: 10.1101/gad.364306 16481475 PMC1369049

[16] Ganster RW Guo Z Shao L Geller DA . Differential effects of TNF-alpha and IFN-gamma on gene transcription mediated by NF-kappaB-Stat1 interactions. J Interferon Cytokine Res Off J Int Soc Interferon Cytokine Res. (2005) 25:707–19. doi: 10.1089/jir.2005.25.707 16318585

[17] Lawrence T Natoli G . Transcriptional regulation of macrophage polarization: enabling diversity with identity. Nat Rev Immunol. (2011) 11:750–61. doi: 10.1038/nri3088 22025054

[18] Platanitis E Decker T . Regulatory networks involving STATs, IRFs, and NFκB in inflammation. Front Immunol. (2018) 9. doi: 10.3389/fimmu.2018.02542 30483250 PMC6242948

[19] Ng CY Whitelock JM Williams H Kim HN Medbury HJ Lord MS . Macrophages bind LDL using heparan sulfate and the perlecan protein core. J Biol Chem. (2021) 296:100520. doi: 10.1016/j.jbc.2021.100520 33684447 PMC8027565

[20] Swart M Troeberg L . Effect of polarization and chronic inflammation on macrophage expression of heparan sulfate proteoglycans and biosynthesis enzymes. J Histochem Cytochem Off J Histochem Soc. (2019) 67:9–27. doi: 10.1369/0022155418798770 30205019 PMC6309031

[21] Viola A Munari F Sánchez-Rodríguez R Scolaro T Castegna A . The metabolic signature of macrophage responses. Front Immunol. (2019) 10:1462. doi: 10.3389/fimmu.2019.01462 31333642 PMC6618143

[22] Murray PJ Wynn TA . Protective and pathogenic functions of macrophage subsets. Nat Rev Immunol. (2011) 11:723–37. doi: 10.1038/nri3073 21997792 PMC3422549

[23] Hillmer EJ Zhang H Li HS Watowich SS . STAT3 signaling in immunity. Cytokine Growth Fact Rev. (2016) 31:1–15. doi: 10.1016/j.cytogfr.2016.05.001 27185365 PMC5050093

[24] Stout RD Suttles J . Functional plasticity of macrophages: reversible adaptation to changing microenvironments. J Leukoc Biol. (2004) 76:509–13. doi: 10.1189/jlb.0504272 15218057 PMC1201486

[25] Porcheray F Viaud S Rimaniol AC Léone C Samah B Dereuddre-Bosquet N . Macrophage activation switching: an asset for the resolution of inflammation. Clin Exp Immunol. (2005) 142:481–9. doi: 10.1111/j.1365-2249.2005.02934.x 16297160 PMC1809537

[26] Murray PJ . Macrophage polarization. Annu Rev Physiol. (2017) 79:541–66. doi: 10.1146/annurev-physiol-022516-034339 27813830

[27] Okabe Y Medzhitov R . Tissue biology perspective on macrophages. Nat Immunol. (2016) 17:9–17. doi: 10.1038/ni.3320 26681457

[28] Iozzo RV Schaefer L . Proteoglycan form and function: a comprehensive nomenclature of proteoglycans. Matrix Biol. (2015) 42:11–55. doi: 10.1016/j.matbio.2015.02.003 25701227 PMC4859157

[29] Theocharis AD Skandalis SS Gialeli C Karamanos NK . Extracellular matrix structure. Adv Drug Delivery Rev. (2016) 97:4–27. doi: 10.1016/j.addr.2015.11.001 26562801

[30] Hu K Shang Z Yang X Zhang Y Cao L . Macrophage polarization and the regulation of bone immunity in bone homeostasis. J Inflammation Res. (2023) 16:3563. doi: 10.2147/JIR.S423819 37636272 PMC10460180

[31] Luo G Li F Li X Wang ZG Zhang B . TNF-α and RANKL promote osteoclastogenesis by upregulating RANK via the NF-κB pathway. Mol Med Rep. (2018) 17:6605–11. doi: 10.3892/mmr.2018.8698 29512766 PMC5928634

[32] Lorenzo J . Cytokines and bone: osteoimmunology. In: Stern PH , editor. Bone Regulators and Osteoporosis Therapy. Springer International Publishing, Cham (2020). p. 177–230. doi: 10.1007/164_2019_346 32006259

[33] Schett G . Osteoimmunology in rheumatic diseases. Arthritis Res Ther. (2009) 11:210. doi: 10.1186/ar2571 19232069 PMC2688223

[34] Yan J Huang C Jiang D Xu Y Zhang Z Wang L . Macrophage polarization and its impact on osteoporosis. In: Kloc M Kubiak JZ Halasa M , editors. Monocytes and Macrophages in Development, Regeneration, and Disease. Springer Nature Switzerland, Cham (2024). p. 291–6. doi: 10.1007/978-3-031-65944-7_11 39406910

[35] Taylan F Costantini A Coles N Pekkinen M Héon E Şıklar Z . Spondyloocular syndrome: novel mutations in XYLT2 gene and expansion of the phenotypic spectrum. J Bone Miner Res. (2016) 31:1577–85. doi: 10.1002/jbmr.2834 26987875

[36] Chouery E Karam R Mrad YN Mehawej C Jalbout NDE Bleik J . Spondyloocular syndrome: a report of an additional family and phenotypic spectrum delineation. Genes. (2023) 14. doi: 10.3390/genes14020497 36833424 PMC9957273

[37] Munns CF Fahiminiya S Poudel N Munteanu MC Majewski J Sillence DO . Homozygosity for frameshift mutations in XYLT2 result in a spondylo-ocular syndrome with bone fragility, cataracts, and hearing defects. Am J Hum Genet. (2015) 96:971. doi: 10.1016/j.ajhg.2015.04.017 26027496 PMC4457947

[38] Götting C Kuhn J Zahn R Brinkmann T Kleesiek K . Molecular cloning and expression of human UDP-d-Xylose:proteoglycan core protein beta-d-xylosyltransferase and its first isoform XT-II. J Mol Biol. (2000) 304:517–28. doi: 10.1006/jmbi.2000.4261 11099377

[39] Roch C Kuhn J Kleesiek K Götting C . Differences in gene expression of human xylosyltransferases and determination of acceptor specificities for various proteoglycans. Biochem Biophys Res Commun. (2010) 391:685–91. doi: 10.1016/j.bbrc.2009.11.121 19944077

[40] Bui C Huber C Tuysuz B Alanay Y Bole-Feysot C Leroy JG . XYLT1 mutations in Desbuquois dysplasia type 2. Am J Hum Genet. (2014) 94:405–14. doi: 10.1016/j.ajhg.2014.01.020 24581741 PMC3951945

[41] Mizumoto S . Defects in biosynthesis of glycosaminoglycans cause hereditary bone, skin, heart, immune, and neurological disorders. Trends Glycosci Glycotechnol. (2018) 30:E67–89. doi: 10.4052/tigg.1812.2E

[42] Yang S He Z Wu T Wang S Dai H . Glycobiology in osteoclast differentiation and function. Bone Res. (2023) 11:55. doi: 10.1038/s41413-023-00293-6 37884496 PMC10603120

[43] Ly TD Wolny M Lindenkamp C Birschmann I Hendig D Knabbe C . The human myofibroblast marker xylosyltransferase-I: a new indicator for macrophage polarization. Biomedicines. (2022) 10:2869. doi: 10.3390/biomedicines10112869 36359389 PMC9687871

[44] Pfaffl MW . A new mathematical model for relative quantification in real-time RT–PCR. Nucleic Acids Res. (2001) 29:e45. doi: 10.1093/nar/29.9.e45 11328886 PMC55695

[45] Love MI Huber W Anders S . Moderated estimation of fold change and dispersion for RNA-seq data with DESeq2. Genome Biol. (2014) 15:550. doi: 10.1186/s13059-014-0550-8 25516281 PMC4302049

[46] Zhu A Ibrahim JG Love MI . Heavy-tailed prior distributions for sequence count data: removing the noise and preserving large differences. Bioinformatics. (2019) 35:2084–92. doi: 10.1093/bioinformatics/bty895 30395178 PMC6581436

[47] Bioconductor . Org.Hs.Eg.Db. Available online at: http://bioconductor.org/packages/org.Hs.eg.db/ (Accessed October 14, 2025).

[48] Ge SX Jung D Yao R . ShinyGO: a graphical gene-set enrichment tool for animals and plants. Bioinformatics. (2020) 36:2628–9. doi: 10.1093/bioinformatics/btz931 31882993 PMC7178415

[49] Schneider CA Rasband WS Eliceiri KW . NIH Image to ImageJ: 25 years of image analysis. Nat Methods. (2012) 9:671–5. doi: 10.1038/nmeth.2089 22930834 PMC5554542

[50] Kleine A Kühle M Kuhn J Ly TD Schmidt V Faust-Hinse I . A novel SPE-UPLC-MS/MS-based assay for the selective, simultaneous quantification of xylosyltransferase-I and -II activity. Biochimie. (2024) 218:127–36. doi: 10.1016/j.biochi.2023.09.008 37689257

[51] Smith PK Krohn RI Hermanson GT Mallia AK Gartner FH Provenzano MD . Measurement of protein using bicinchoninic acid. Anal Biochem. (1985) 150:76–85. doi: 10.1016/0003-2697(85)90442-7 3843705

[52] Yao Y Cai X Ren F Ye Y Wang F Zheng C . The macrophage-osteoclast axis in osteoimmunity and osteo-related diseases. Front Immunol. (2021) 12:664871. doi: 10.3389/fimmu.2021.664871 33868316 PMC8044404

[53] Sun Y Li J Xie X Gu F Sui Z Zhang K . Macrophage-osteoclast associations: Origin, polarization, and subgroups. Front Immunol. (2021) 12. doi: 10.3389/fimmu.2021.778078 34925351 PMC8672114

[54] Wang W Liu H Liu T Yang H He F . Insights into the role of macrophage polarization in the pathogenesis of osteoporosis. Oxid Med Cell Longev. (2022) 2022:2485959. doi: 10.1155/2022/2485959 35707276 PMC9192196

[55] Ferencz B Condac E Poudel N Munteanu MC Sivasami P Choudhury B . Xylosyltransferase 2 deficiency and organ homeostasis. Glycoconj J. (2020) 37:755–65. doi: 10.1007/s10719-020-09945-9 32965647 PMC9248025

[56] Kleine A Kühle M Ly TD Schmidt V Faust-Hinse I Knabbe C . Xylosyltransferase-deficiency in human dermal fibroblasts induces compensatory myofibroblast differentiation and long-term ECM reduction. Biomedicines. (2024) 12:572. doi: 10.3390/biomedicines12030572 38540185 PMC10967791

[57] Rajaram M Ni B Carlson T Schlesinger L . Mannose receptor (CD206)-mediated signaling in human macrophages in the context of tuberculosis (INC7P.418). J Immunol. (2014) 192:186.19. doi: 10.4049/jimmunol.192.Supp.186.19

[58] Hickman E Smyth T Cobos-Uribe C Immormino R Rebuli ME Moran T . Expanded characterization of *in vitro* polarized M0, M1, and M2 human monocyte-derived macrophages: Bioenergetic and secreted mediator profiles. PloS One. (2023) 18:e0279037. doi: 10.1371/journal.pone.0279037 36862675 PMC9980743

[59] Aota K Yamanoi T Kani K Ono S Momota Y Azuma M . Inhibition of JAK-STAT signaling by baricitinib reduces interferon-γ-induced CXCL10 production in human salivary gland ductal cells. Inflammation. (2021) 44:206–16. doi: 10.1007/s10753-020-01322-w 32772240

[60] Soejima K Rollins BJ . A functional IFN-γ-inducible protein-10/CXCL10-specific receptor expressed by epithelial and endothelial cells that is neither CXCR3 nor glycosaminoglycan. J Immunol. (2001) 167:6576–82. doi: 10.4049/jimmunol.167.11.6576 11714827

[61] Brownell J Bruckner J Wagoner J Thomas E Loo YM Gale M . Direct, interferon-independent activation of the CXCL10 promoter by NF-κB and interferon regulatory factor 3 during hepatitis C virus infection. J Virol. (2014) 88:1582–90. doi: 10.1128/JVI.02007-13 24257594 PMC3911583

[62] Ciesielska A Matyjek M Kwiatkowska K . TLR4 and CD14 trafficking and its influence on LPS-induced pro-inflammatory signaling. Cell Mol Life Sci. (2021) 78:1233–61. doi: 10.1007/s00018-020-03656-y 33057840 PMC7904555

[63] Pivotal Advance: Endogenous Pathway to SIRS, Sepsis, and Related Conditions | Journal of Leukocyte Biology | Oxford Academic. Available online at: https://academic.oup.com/jleukbio/article-abstract/82/2/282/6975462?redirectedFrom=fulltext&login=false (Accessed October 14, 2025). 10.1189/jlb.1206752 17495051

[64] Roedig H Nastase MV Frey H Moreth K Zeng-Brouwers J Poluzzi C . Biglycan is a new high-affinity ligand for CD14 in macrophages. Matrix Biol. (2019) 77:4–22. doi: 10.1016/j.matbio.2018.05.006 29777767

[65] Francos-Quijorna I Sánchez-Petidier M Burnside ER Badea SR Torres-Espin A Marshall L . Chondroitin sulfate proteoglycans prevent immune cell phenotypic conversion and inflammation resolution via TLR4 in rodent models of spinal cord injury. Nat Commun. (2022) 13:2933. doi: 10.1038/s41467-022-30467-5 35614038 PMC9133109

[66] Oeckinghaus A Ghosh S . The NF-κB family of transcription factors and its regulation. Cold Spring Harb Perspect Biol. (2009) 1:a000034. doi: 10.1101/cshperspect.a000034 20066092 PMC2773619

[67] Hinz M Scheidereit C . The IκB kinase complex in NF-κB regulation and beyond. EMBO Rep. (2014) 15:46–61. doi: 10.1002/embr.201337983 24375677 PMC4303448

[68] Alanazi A Nagi MN Alhareth DY Al-Hamamah MA Mahmoud MA Ahmad SF . Crosstalk of TNF-α, IFN-γ, NF-kB, STAT1 and redox signaling in lipopolysaccharide/d-galactosamine/dimethylsulfoxide-induced fulminant hepatic failure in mice. Saudi Pharm J. (2023) 31:370–81. doi: 10.1016/j.jsps.2023.01.005 37026046 PMC10071328

[69] Hu X Li J Fu M Zhao X Wang W . The JAK/STAT signaling pathway: From bench to clinic. Signal Transd Targ Ther. (2021) 6:402. doi: 10.1038/s41392-021-00791-1 34824210 PMC8617206

[70] Martinez FO Helming L Gordon S . Alternative activation of macrophages: An immunologic functional perspective. Annu Rev Immunol. (2009) 27:451–83. doi: 10.1146/annurev.immunol.021908.132532 19105661

[71] Liang YB Tang H Chen ZB Zeng LJ Wu JG Yang W . Downregulated SOCS1 expression activates the JAK1/STAT1 pathway and promotes polarization of macrophages into M1 type. Mol Med Rep. (2017) 16:6405–11. doi: 10.3892/mmr.2017.7384 28901399

[72] Hong F Jaruga B Kim WH Radaeva S El-Assal ON Tian Z . Opposing roles of STAT1 and STAT3 in T cell–mediated hepatitis: Regulation by SOCS. J Clin Invest. (2002) 110:1503–13. doi: 10.1172/JCI15841 12438448 PMC151811

[73] Zhao J Yu H Liu Y Gibson SA Yan Z Xu X . Protective effect of suppressing STAT3 activity in LPS-induced acute lung injury. Am J Physiol Lung Cell Mol Physiol. (2016) 311:L868–80. doi: 10.1152/ajplung.00281.2016 27638904 PMC5130536

[74] Samad MA Ahmad I Hasan A Alhashmi MH Ayub A Al-Abbasi FA . STAT3 signaling pathway in health and disease. MedComm. (2025) 6:e70152. doi: 10.1002/mco2.70152 40166646 PMC11955304

[75] Rawat R Rainey GJ Thompson CD Frazier-Jessen MR Brown RT Nordan RP . Constitutive activation of STAT3 is associated with the acquisition of an interleukin 6-independent phenotype by murine plasmacytomas and hybridomas. Blood. (2000) 96:3514–21. doi: 10.1182/blood.v96.10.3514 11071649

[76] Silva AD Hwang J Marciel MP Bellis SL . The pro-inflammatory cytokines IL-1β and IL-6 promote upregulation of the ST6GAL1 sialyltransferase in pancreatic cancer cells. J Biol Chem. (2024) 300:107752. doi: 10.1016/j.jbc.2024.107752 39260693 PMC11470512

[77] Wang W Lopez McDonald MC Kim C Ma M Pan Z( Kaufmann C . The complementary roles of STAT3 and STAT1 in cancer biology: Insights into tumor pathogenesis and therapeutic strategies. Front Immunol. (2023) 14. doi: 10.3389/fimmu.2023.1265818 38022653 PMC10663227

[78] Wight TN . A role for proteoglycans in vascular disease. Matrix Biol J Int Soc Matrix Biol. (2018) 71–72:396–420. doi: 10.1016/j.matbio.2018.02.019 29499356 PMC6110991

[79] Ma M Jiang W Zhou R . DAMPs and DAMP-sensing receptors in inflammation and diseases. Immunity. (2024) 57:752–71. doi: 10.1016/j.immuni.2024.03.002 38599169

[80] Lin H Xiong W Fu L Yi J Yang J . Damage-associated molecular patterns (DAMPs) in diseases: Implications for therapy. Mol BioMed. (2025) 6:60. doi: 10.1186/s43556-025-00305-3 40877572 PMC12394712

[81] Li H Sun S . Protein aggregation in the ER: Calm behind the storm. Cells. (2021) 10:3337. doi: 10.3390/cells10123337 34943844 PMC8699410

[82] Adams CJ Kopp MC Larburu N Nowak PR Ali MMU . Structure and molecular mechanism of ER stress signaling by the unfolded protein response signal activator IRE1. Front Mol Biosci. (2019) 6:11. doi: 10.3389/fmolb.2019.00011 30931312 PMC6423427

[83] Sprooten J Garg AD . Type I interferons and endoplasmic reticulum stress in health and disease. Int Rev Cell Mol Biol. (2020) 350:63–118. doi: 10.1016/bs.ircmb.2019.10.004 32138904 PMC7104985

[84] Babadei O Strobl B Müller M Decker T . Transcriptional control of interferon-stimulated genes. J Biol Chem. (2024) 300:107771. doi: 10.1016/j.jbc.2024.107771 39276937 PMC11489399

[85] Schoggins JW . Interferon-stimulated genes: What do they all do? Annu Rev Virol. (2019) 6:567–84. doi: 10.1146/annurev-virology-092818-015756 31283436

[86] Legrand A Dahoui C De La Myre Mory C Noy K Guiguettaz L Versapuech M . SAMD9L acts as an antiviral factor against HIV-1 and primate lentiviruses by restricting viral and cellular translation. PloS Biol. (2024) 22:e3002696. doi: 10.1371/journal.pbio.3002696 38959200 PMC11221667

[87] Wicherska-Pawłowska K Wróbel T Rybka J . Toll-like receptors (TLRs), NOD-like receptors (NLRs), and RIG-I-like receptors (RLRs) in innate immunity. TLRs, NLRs, and RLRs ligands as immunotherapeutic agents for hematopoietic diseases. Int J Mol Sci. (2021) 22:13397. doi: 10.3390/ijms222413397 34948194 PMC8704656

[88] Sivasami P Poudel N Munteanu MC Hudson J Lovern P Liu L . Adipose tissue loss and lipodystrophy in xylosyltransferase II deficient mice. Int J Obes 2005. (2019) 43:1783–94. doi: 10.1038/s41366-019-0324-1 30778123 PMC7067554

