# Supplementary material for: Xylosyltransferase-II deficiency rewires innate immune signaling and destabilizes polarization in human macrophages
Source: Front Immunol. 2026 Jun 26;17:1850248. doi: 10.3389/fimmu.2026.1850248 (PMC13349761; doi:10.3389/fimmu.2026.1850248)
Supplement: Supplementary file 4 [file DataSheet1.pdf]

Supplementary Material

Table S1 Sequences of primers for qRT-PCR

Target gene	Sequence (5'-3')
hß2M	TGTGCTCGCGCTACTCTCTCTT<br>CGGATGGATGAAACCCAGACA
hCD163	ACAAAAAGCCACAACAGGTCG<br>GGTATCTTAAAGGCTCACTGGGT
hCD206	TGCTACTGAACCCCCACAAC<br>ACCAGAGAGGAACCCATTCG
hCXCL10	AAAGCAGTTAGCAAGGAAAGGTCT<br>AGGGAAGTGATGGGAGAGGCAG
hIL1ß	ACAGATGAAGTGCTCCTTCCA<br>GTCGGAGATTCGTAGCTGGA
hIL6	ACAGCCACTCACCTCTTCAG<br>GTGCCTCTTTGCTGCTTTCAC
hIκBα	TTTTGGTGTCCTTGGGTGCT<br>GTCATCATAGGGCAGCTCGT
hNF-κB1	ACCCTCAGGTCAAAATCTGCAA<br>AATAGGCAAGGTCAGGGTGC
hNF-κB2	GGGGGCATCAAACCTGAAGA<br>ACCTCTTCCTTGTCTTCCACC
hNF-κB p65/RelA	AGCTTGTAGGAAAGGACTGCC<br>CCACGCTGCTCTTCTTGGAA
hRPL13	CGGAAGGTGGTGGTCGTA<br>CTCGGGAAGGGTTGGTGT
hSDHA	AACTCGCTCTTGGACCTG<br>GAGTCGCAGTTCCGATGT
hTLR4	CCCTGAGGCATTTAGGCAGCTA<br>AGGTAGAGAGGTGGCTTAGGCT
hTNFα	CCCCAGGGACCTCTCTCTAA<br>CTCAGCTTGAGGGTTTGCTAC
hXYLT1	GAAGCCGTGGTGAATCAG<br>CGGTCAGCAAGGAAGTAG
hXYLT2	ACACAGATGACCCGCTTGTGG<br>TTGGTGACCCGCAGGTTGTTG

Table S2. qRT-PCR protocol for gene expression analysis

Step	Time [s]	Temperature [°C]	Cycle
Preincubation	300	95	1
Denaturation	10	95	40
Annealing	15	T_a (primer-specific)	
Elongation	20	72	
Detection	-	72	
Melting curve	5	95	1
	60		
Cooling	60	65	1

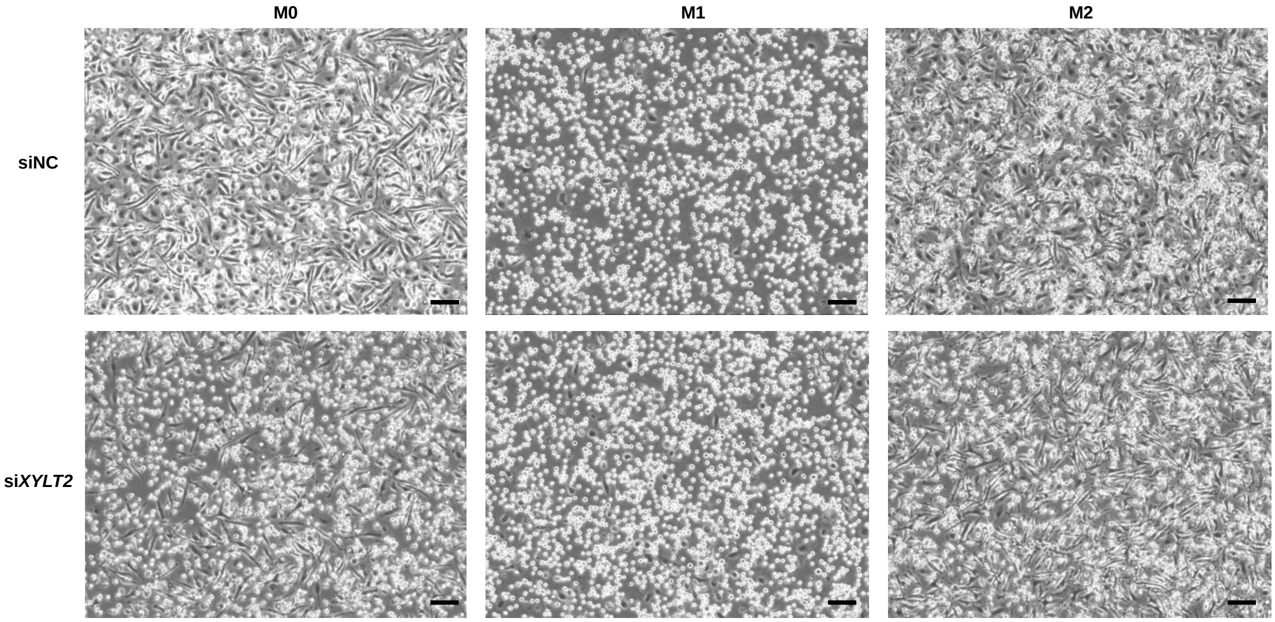

Figure S 1: Morphology of polarized macrophages after siRNA-mediated *XYLT2* knockdown. Negatively selected monocytes were differentiated into macrophages using M-CSF. On day 5, macrophages were treated with a non-targeting negative control siRNA (siNC) or a siRNA targeting *XYLT2* (si*XYLT2*). On day 6, macrophages were stimulated with IFNγ/LPS (M1), IL4 (M2), or with no additive (M0). Cells were analyzed after a polarization time of 48 h. Exemplary images of macrophage morphology are shown. Scale bar = 100 µm.

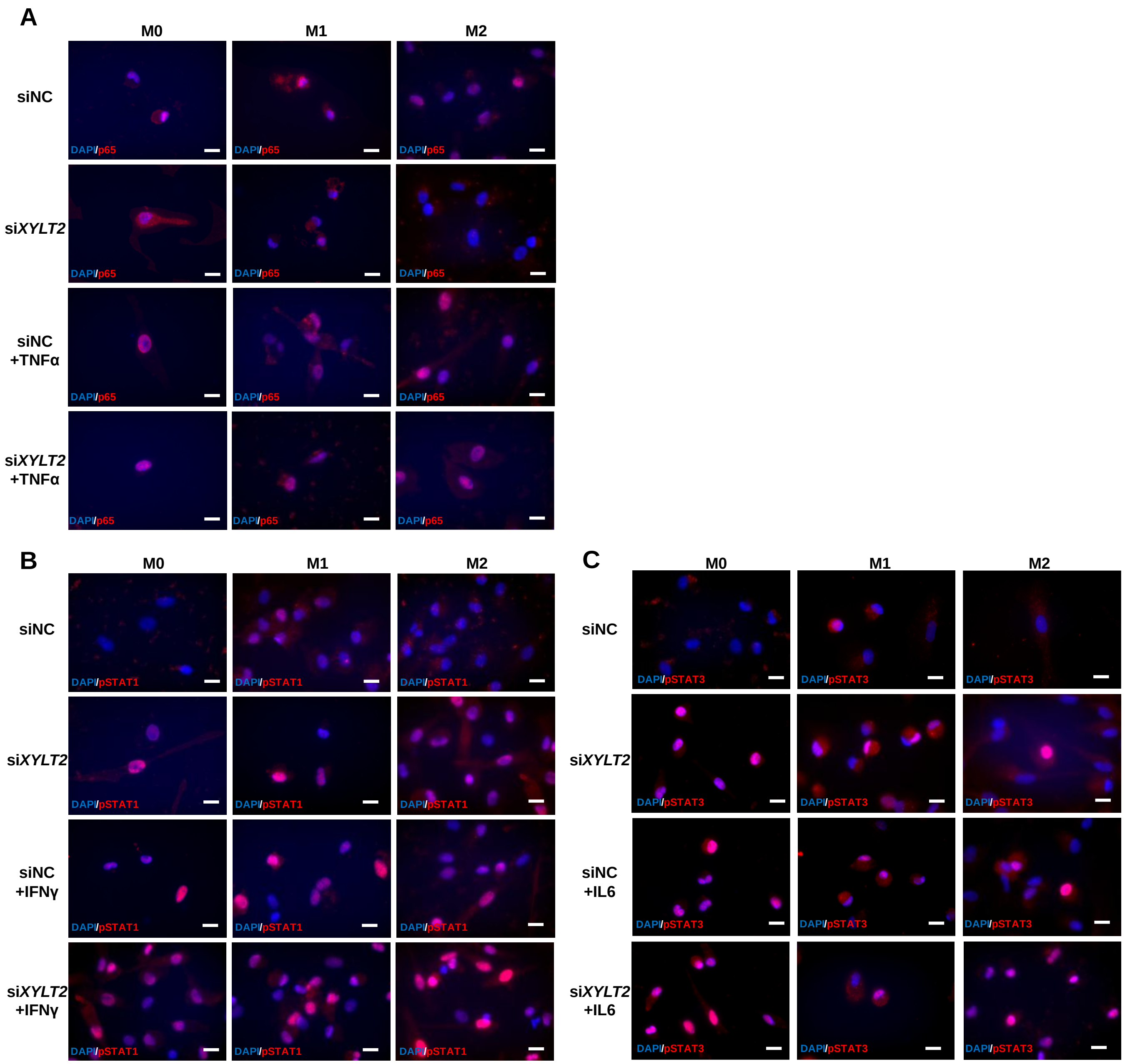

Figure S 2: Immunofluorescence analysis of NF-κB p65, pSTAT1, and pSTAT3 in polarized macrophages after siRNA-mediated *XYLT2* knockdown. Negatively selected monocytes were differentiated into macrophages using M-CSF. On day 5, macrophages were treated with a non-targeting negative control siRNA (siNC) or a siRNA targeting *XYLT2* (si*XYLT2*). On day 6, macrophages were stimulated with IFNγ/LPS (M1), IL4 (M2), or without additional stimuli (M0) and polarized for 48 h. Where indicated, macrophages were additionally stimulated with TNFα (20 ng/mL), IFNγ (50 ng/mL), or IL6 (50 ng/mL) for 30 min prior to fixation to assess stimulus-induced signaling responses. Exemplary images of NF-κB p65/RelA **(A)**, pSTAT1 **(B)** and pSTAT3 **(C)** shown. Nuclei were counterstained with DAPI. Scale bar = 10 µm.

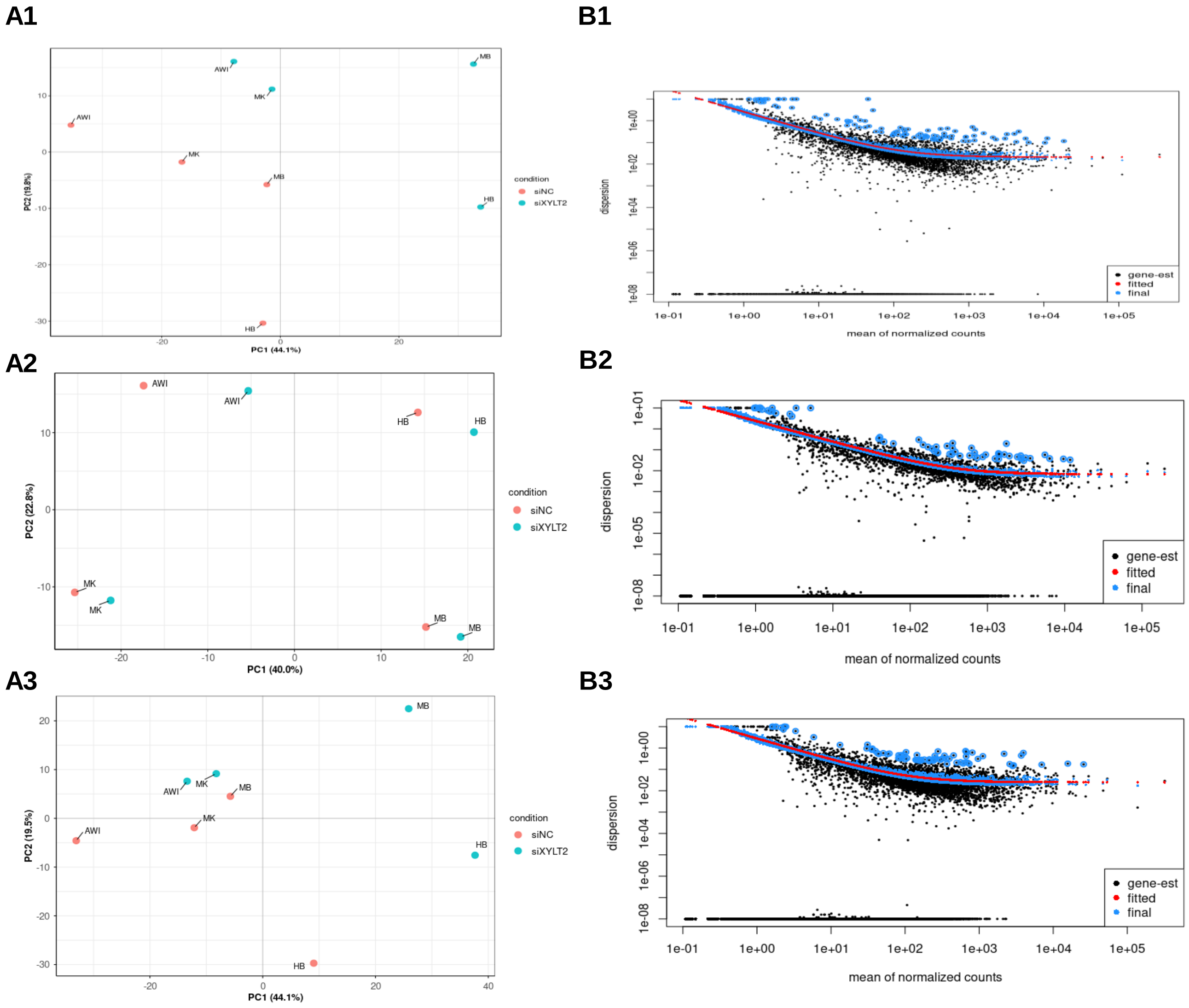

Figure S 3: Principal component analysis and dispersion estimation for analysis of differentially expressed genes in polarized macrophages after siRNA-mediated *XYLT2* knockdown. Negatively selected monocytes were differentiated into macrophages using M-CSF. On day 5, macrophages were treated with a non-targeting negative control siRNA (siNC) or a siRNA targeting *XYLT2* (si*XYLT2*). On day 6, macrophages were stimulated with IFNγ/LPS (M1), IL4 (M2), or with no additive (M0). Cells were harvested for mRNA-Seq (n =4) after a polarization time of 48 h. Principal component analysis (PCA) plots **(A)** and dispersion estimation plots **(B)** are shown for M0 **(1)**, M1 **(2)** and M2 **(3)** macrophages. PCA was performed on variance-stabilizing transformed (VST) counts. Each data point represents one donor (n = 4 per condition); siNC samples are shown in red, si*XYLT2* samples in turquoise. The percentage of variance explained by each principal component is indicated on the respective axis. Dispersion plots show gene-wise dispersion estimates (black), the fitted mean-dispersion relationship (red), and final shrunken dispersion estimates (blue circles).

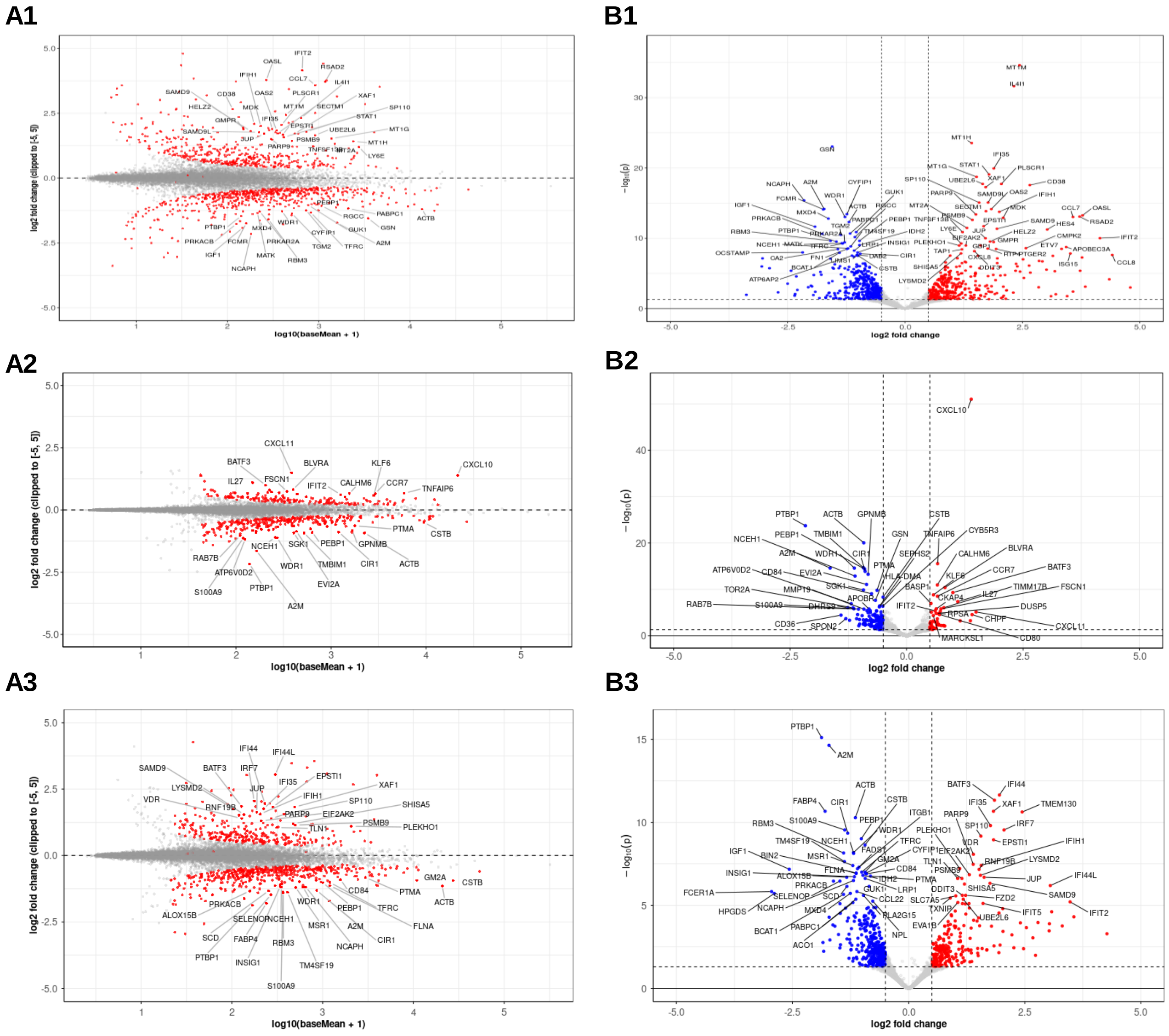

Figure S 4: MA plots and volcano plots for analysis of differentially expressed genes in polarized macrophages after siRNA-mediated *XYLT2* knockdown Negatively selected monocytes were differentiated into macrophages using M-CSF. On day 5, macrophages were treated with a non-targeting negative control siRNA (siNC) or a siRNA targeting *XYLT2* (si*XYLT2*). On day 6, macrophages were stimulated with IFNγ/LPS (M1), IL4 (M2), or with no additive (M0). Cells were harvested for mRNA-Seq (n =4) after a polarization time of 48 h. MA plots **(A)** and volcano plots **(B)** are shown for M0 **(1)**, M1 **(2)**, and M2 **(3)** macrophages following siRNA-mediated *XYLT2* knockdown relative to siNC controls. In MA plots, each point represents one gene; log2 fold changes (apeglm-shrunken) are plotted against the mean of normalized counts. Red points indicate significantly differentially expressed genes (padj < 0.05, |LFC| ≥ 0.5); grey points indicate non-significant genes. LFC values are clipped to [−5, 5] for visualization purposes. In volcano plots, the −log10(adjusted p-value) is plotted against the log2 fold change. Red points indicate significantly upregulated genes, blue points indicate significantly downregulated genes; grey points are non-significant. Dashed lines indicate the significance thresholds (padj < 0.05; |LFC| ≥ 0.5). Selected genes of biological interest are labeled.
